# Renal functional, transcriptome, and methylome adaptations in pregnant Sprague Dawley and Brown Norway rats

**DOI:** 10.1371/journal.pone.0269792

**Published:** 2022-06-16

**Authors:** Zhong Chen, Charles Wang, Arlin Blood, Shannon Bragg, Eugenia Mata-Greenwood

**Affiliations:** 1 Center for Genomics, School of Medicine, Loma Linda University, Loma Linda, CA, United States of America; 2 Lawrence D. Longo Center for Perinatal Biology, School of Medicine, Loma Linda University, Loma Linda, CA, United States of America; 3 Department of Pediatrics, School of Medicine, Loma Linda University, Loma Linda, CA, United States of America; Max Delbruck Centrum fur Molekulare Medizin Berlin Buch, GERMANY

## Abstract

Pregnancy induces maternal renal adaptations that include increased glomerular filtration rate and renal blood flow which can be compromised in obstetrical complications such as preeclampsia. Brown Norway (BN) rat pregnancies are characterized by placental insufficiency, maternal hypertension, and proteinuria. We hypothesized that BN pregnancies would show renal functional, anatomical, or molecular features of preeclampsia. We used the Sprague-Dawley (CD) rat as a model of normal pregnancy. Pregnancy increased the glomerular filtration rate by 50% in CD rats and 12.2% in BN rats compared to non-pregnancy, and induced proteinuria only in BN rats. BN pregnancies showed a decrease in maternal plasma calcitriol levels, which correlated with renal downregulation of 1-alpha hydroxylase and upregulation of 24-hydroxylase. RNA sequencing revealed that pregnancy induced 297 differentially expressed genes (DEGs) in CD rats and 174 DEGs in BN rats, indicating a 70% increased response to pregnancy in CD compared to BN rats. Pregnancy induced activation of innate immune pathways such as ‘Role of Pattern Recognition Receptors’, and ‘Interferon signaling’ with interferon regulatory factor 7 as a common upregulated upstream factor in both rat strains. Comparison of rat strain transcriptomic profiles revealed 475 DEGs at non-pregnancy and 569 DEGs at pregnancy with 205 DEGs shared at non-pregnancy (36%), indicating that pregnancy interacted with rat strain in regulating 64% of the DEGs. Pathway analysis revealed that pregnancy induced a switch in renal transcriptomics in BN rats from ‘inhibition of renal damage’ to ‘acute phase reaction’, ‘recruitment of immune cells’ and ‘inhibition of 1,25-(OH)_2_-vitamin D synthesis’. Key upstream regulators included peroxisome-proliferator-activated receptor alpha (PPARA), platelet-derived growth factor B dimer (PDGF-BB), and NF-kB p65 (RELA). DNA methylome profiling by reduced representation bisulfite sequencing studies revealed that the DEGs did not correlate with changes in promoter methylation. In sum, BN rat kidneys respond to pregnancy-specific signals with an increase in pro-inflammatory gene networks and alteration of metabolic pathways including vitamin D deficiency in association with mild proteinuria and blunted GFR increase. However, the lack of glomerular endotheliosis and mild hypertension/proteinuria in pregnant BN rats limits the relevance of this rat strain for preeclampsia research.

## Introduction

Preeclampsia is a leading cause of pregnancy related morbidity and mortality and affects between 3–7% of pregnancies in the United States [1, 2]. Preeclampsia is a complex disease of unknown etiology consisting of de novo hypertension after week 20 of pregnancy that is often accompanied by proteinuria [1, 2]. Although the etiology of this disease remains unknown, it is widely accepted that placental insufficiency in correlation with, or potentially due to, decreased remodeling of maternal uterine arteries is a pivotal event that precedes the onset of this disease [3]. Women with renal insufficiency have an increased risk of developing preeclampsia [4–6], which indicates an important contribution of this organ in the development of this disease. Glomerular endotheliosis with loss of fenestrations is a characteristic of preeclampsia-related temporary renal damage. [5, 6]. Nevertheless, the molecular mechanisms responsible for the renal adaptations observed in normal and preeclampsia pregnancies remain poorly understood, although some key molecules such as nephrin, complement factors, and vascular endothelial growth factor (VEGF) have been shown to be altered in preeclampsia kidneys [7–9]. In addition, dysregulation of vitamin D metabolism, specifically decreased circulatory levels of vitamin D metabolites, is yet another key characteristic of preeclampsia [10, 11]. Vitamin D deficiency/insufficiency in preeclampsia is likely due to both exogenous (nutritional/lifestyle) and metabolic/endogenous factors [12, 13]. Because of the multiple systems involved in this disease (placental-maternal vascular system, endocrine, immune, renal and hepatic systems), the use of rat models of pregnancy health and disease can be useful to uncover key physiological and molecular pathways specific to pregnancy.

We have previously shown that Brown Norway rat pregnancies are characterized by shallow trophoblast remodeling of uterine arteries and placental insufficiency [14]. These early-onset events are followed by moderate hypertension and proteinuria [15], all of which resolve after delivery. Of interest, Brown Norway rats show a pregnancy specific vitamin D deficiency in plasma levels of 1,25-(OH)_2_-D with normal levels of 25-OH-D [16]. In contrast, other rat strains such as Sprague-Dawley and Lewis demonstrate a pregnancy-induced 2-fold increase of circulating 1,25-(OH)_2_-D and a 40% decrease in 25-OH-D levels with pregnancy [16]. This is of interest as preeclampsia that is associated with FGR shows a significant decrease in maternal plasma levels of 1,25-(OH)_2_-D with lesser decreases in 25-OH-D [13, 17]. The kidney is a key organ in regulating the circulating levels of 1,25-(OH)_2_-D in all mammals [18]. Therefore, we hypothesized that Brown Norway rat pregnancies would show renal functional (decreased glomerular filtration rate, proteinuria), anatomical (glomerular endotheliosis), and/or molecular features (i.e. increased complement factors) of preeclampsia.

## Materials and methods

### Animals

Two-month old Brown Norway (BN) and Sprague Dawley (CD) male and female rats were obtained from Charles River Laboratories (Cambridge, MA) and housed at the Animal Research Facility of Loma Linda University under conditions of 14h light, 10h darkness, room temperature of 20°C, and relative humidity of 30–60%. Rats were fed standard rat chow and tap water *ad libitum*. Experimental protocols were in compliance with the Animal Welfare Act and were approved by the Institutional Animal Care and Use Committee of Loma Linda University.

To study renal adaptations to pregnancy in BN and CD rats, pregnant (day 18, P18) and age-matched non-pregnant (NP) rats were studied. Female rats were housed in single cages. Rats were bred by overnight monogamous pairing of a female with a strain-matched male. Two sets of rat cohorts, each composed of 20 female rats (5 CD-NP, 5 CD-P18, 5 BN-NP, and 5 BN-P18), were used: 1) set A for renal transcriptomic and epigenomics, renal morphology, proteinuria, and plasma vitamin D metabolite assays, and 2) set B for glomerular filtration rate and cardiovascular parameter assays (S1 Fig). To collect heparinized plasma and fresh tissues, the rats were anesthetized with 2% isoflurane and blood was withdrawn directly from the beating heart using lithium heparin tubes followed by heart/lung block removal. Fresh kidneys were quickly removed, cleaned, and stored in 3% glutaraldehyde in 0.1M cacodylate buffer for electron microscopy analysis or snap-frozen in liquid nitrogen for transcriptomic and epigenomic studies. Plasma, urine, and snap-frozen tissues (maternal kidneys and placentas) were stored at -70°C until further analysis. Rat pregnancy parameters such as litter size, fetal weight (average/litter) and placental weight (average/litter) were also obtained.

### Renal functional assays

GFR was measured in urethane-anesthetized rat using FITC inulin, as previously described [19]. Previous to this assay, mean blood pressure and heart rates were determined through a femoral artery catheter transducer, as previously described [15]. Afterwards, inulin-FITC (5 mg/mL) containing 40 mg/mL BSA in saline was infused at a rate of 1 mL/hr per 100 g of body weight. Urine was collected via a heat-flared cannula inserted in the bladder. After obtaining a stable urine flow rate (~20 μL/min), a 20 min urine aliquot was collected followed by a 0.5 mL blood sample. A second 20 min urine aliquot was then collected, followed by rat euthanization via thoracotomy. Both kidneys were removed, dried, and weighed. Inulin-FITC was measured in both plasma and urine aliquots using a Tekon Fluorometer, as previously described. Urine Flow Rate (UVR; mL/min): [volume of urine collected] ÷ [time of collection] and Glomerular Filtration Rate (GFR; mL/min): [Urine inulin concentration x UVR ÷ [Plasma inulin concentration]) were calculated for each rat before calculating group averages.

To determine maternal proteinuria, rats were placed in metabolic cages and 2 mL of urine was collected under fasting conditions. Urine collections were performed the day before necropsy and tissue collection. Urinary excretion of protein was determined using the Quantichrom™ protein-to-creatinine ratio kit (Bioassay Systems, Hayward, CA), according to the manufacturer’s protocols. Urinary Protein-to-Creatinine ratio is reported as mg of protein per g of creatinine. Mild proteinuria is designated as 50–300 mg/g and severe proteinuria is >300 g/g.

Renal homeostasis correlates with the regulation of plasma 1,25-(OH)_2_-D levels; although during pregnancy the placenta also contributes to the biosynthesis of this vitamin D metabolite [16–18]. Both total 25-OH-D and 1,25-(OH)_2_-D were analyzed by commercially available ELISAs (Immunodiagnostic systems, UK) according to the manufacturer’s protocol. We have previously validated these kits using LC/MS [16]. Normal ranges are designated as 50–200 nM for 25-OH-D and 50–175 pM for 1,25-(OH)_2_-D.

### Electron microscopy of glomeruli

Maternal kidneys (BN-NP, BN-P18, CD-NP, CD-P18, 3/group) were processed and analyzed by the Center for Microscopy Services of the University of California, Riverside. At least 6 glomeruli endothelial cells from each kidney (n = 12, 3/group) were photographed and studied for structural features of endotheliosis with advice from the LLU pathology department.

### Renal transcriptome analysis

To study the renal mRNA changes due to pregnancy, we performed RNA-sequencing in kidney cross-sections containing 2/3 cortex and 1/3 medulla from age-matched CD and BN rat strains at either pregnancy day 18 or non-pregnancy. A 40 mg representative renal was used to isolate total RNA from each rat. All RNA samples were tested for purity and integrity, and all samples had a RIN higher than 8.

#### RNA-Seq library construction

The Ovation^®^ Rat RNA-Seq System 1–16 (NuGEN Technologies, San Carlos, CA) was used per manufacturer’s instructions to construct RNA-seq libraries. 100 ng of total RNA was used as input. First and second strands of cDNA were synthesized from total RNA (100 ng) spiked with 1 μl of 1:500 diluted ERCC ExFold RNA Spike-In Mix 2 (Life Technologies, Carlsbad, CA) at a final concentration of 1%. Following primer annealing and cDNA synthesis, end-repair, adaptor index ligation, and strand selection was conducted. Barcodes with unique indices was used per sample for multiplexing. Ribosomal RNA depletion was performed by using custom InDA-C primer mixture SS5 V8 for rat. Finally, libraries were amplified for 13 cycles (Mastercycler^®^ pro, Eppendorf, Hamburg, Germany), and purified with Agencourt XP beads (Beckman Coulter, Indianapolis, IN).

#### Library quantification, quality control (QC) and sequencing

The final amplified libraries were purified using Agencourt XP beads and quantified using the Qubit dsDNA HS Kit on Qubit 3.0 Fluorometer (Life Technologies, Carlsbad, CA). Quality and peak size was determined with the D1000 ScreenTape on Agilent 2200 TapeStation (Agilent Technologies, Santa Clara, CA). Final libraries were diluted to 4 nM and libraries of different indices were pooled for sequencing together in equimolar amounts. Each pooled library at a final concentration of about 2.1 pM was clustered using Illumina’s NextSeq 550 FlowCell and sequenced using NextSeq 550 high output kit (Illumina, Inc., San Diego, CA). Single reads with 75bp (RRBS) and 100bp (RNA-seq) were generated.

#### RNA-seq data analysis

For RNA-seq data, we adopted the pipelines used in our recent publications for mRNA-seq data visualization, which integrated FastQC and Cutadapt processes [20], alignment (STAR) [21], reads quantification (HTSeq-count) [22], and differentially expressed gene (DEG) analysis for mRNA-seq data analyses with edgeR package [23]. Briefly, the RNA-seq raw fastq data were first trimmed using Trimmomatic. The trimmed reads were aligned to the rat reference genome (NCBI Rnor6.0) with STAR [21] with default parameter settings. The aligned bam files were then processed using HTSeq-Count for gene quantification. Genes with CPM ≥ 1 in all samples were used for DEG analysis. The differentially expressed genes (DEGs) were identified with FDR < 0.1, and fold change (FC) > 1.25.

#### Validation of RNA-sequencing data by quantitative real-time PCR

Transcriptomics data was validated in a larger set of subjects (n = 5/group, 20 total). SYBR-green exon-spanning primers (S1 Table) were designed and confirmed by standardized efficiency testing and PCR product sequencing. Total RNA was isolated from kidney sections with TRIzol reagent (Life Technologies) and subjected to reverse transcription using the Quantitect RT (Qiagen, Ca). The relative mRNA abundance of selected genes was determined by real time PCR using the QuantiTect SYBR green supermix (Qiagen, Ca) on the CFX Connect™ system (Bio-Rad) as previously described [17]. β-actin (*Actb*) was used an internal control; and negative controls (no template) were used in every run. The relative expression of each gene of interest was calculated by 2^-ΔΔCt^ method and expressed as fold of the Sprague-Dawley non-pregnant average used as control.

### Renal methylome analysis

#### DNA methylation and Reduced Representation Bisulfite Sequencing (RRBS) library construction

We processed 100 ng of isolated gDNA to generate RRBS DNA library using the Ovation^®^ RRBS Methyl-Seq System (NuGEN Technologies, San Carlos, CA) according to the manufacturer’s protocol. Briefly, the methylation insensitive MspI enzyme, which cuts the DNA at CCGG sites, was used to digest gDNA into fragments. The fragments were directly subjected to end blunting and phosphorylation in preparation for ligation to a methylated adapter with a single-base T overhang. A unique index was used per sample for multiplexing. The ligation products were final repaired in a thermal cycler under the program (60°C– 10 min, 70°C– 10 min, hold at 4°C). The product of the final repair reaction was used for bisulfite conversion using kit QIAGEN EpiTect Fast DNA Bisulfite Kit according to Qiagen’s protocol. Bisulfite-converted DNA was then amplified (Mastercycler^®^ pro, Eppendorf, Hamburg, Germany) and bead-purified with Agencourt XP Beads.

#### RRBS data analysis

For the RRBS data, we used a pipeline that integrates the read quality and adapter trimming process [20], NuGEN diversity trimming and N6 de-duplicate scripts, alignment (Bismark) [24], and differential methylation analysis using MethylKit [25]. This pipeline facilitates a rapid transition from sequencing reads to a fully annotated CpG methylation report for biological interpretation. Briefly, the RRBS raw fastq data were first trimmed using Trim Galore 0.4.5. The rat genome NCBI Rnor6.0 was used as a reference genome. Reads were aligned to the rat reference genome with Bismark v0.16.334 by default parameter settings. The methylation call files including the location of each CpG sites and the methylation percentage were generated by the bismark_methylation_extractor function. The aligned SAM files were further processed through MethylKit to generate differential CpG methylation profiling. CpG sites with a minimum coverage of 10 reads in all samples were used for follow-up analysis.

#### Validation of RRBS-methylomic data

We used a methyl-DNA immunoprecipitation (MeDIP) kit (Abcam) to validate differentially methylated regions (DMRs). After integration of DEGs and DMRs, 2 genes, metallothionein 1 (*Mt1*) and an organic cation transporter *Slc22a13* were selected to study the DMRs via MeDIP. SYBR-green primers were designed for each DMR to have an efficiency near 100% (S1 Table). MeDIP was performed according to the manufacturer’s protocol. Briefly, genomic DNA was isolated from each rat kidney (n = 5 per group) and sheared for 20 seconds 3x at 25% amplitude to generate DNA fragments of 200–600 bp. Then, 1 ug sheared gDNA was immunoprecipitated with a methylcytosine specific antibody overnight, and the immunoprecipitated was captured by beads, washed, and released from beads by proteinase K digestion. Both MeDIP and an aliquot of sheared gDNA (input DNA) were purified and used for real time PCR. Methylation was expressed as % methylated/input DNA difference and compared to non-pregnant Sprague-Dawley used as the control.

### Pathway/network analysis

We analyzed the annotated gene datasets obtained with stringer cutoffs of FC>1.5 and FDR <0.05 using the Ingenuity Pathway Analysis Program (Ingenuity Systems, Redwood City, CA), as previously described [26]. The pathways identified were ranked based on the ratio of the number of molecules in a given pathway that are altered in the present dataset vs. the total known molecules that constitute the pathways. The ratio was used to measure the number of genes overlap and p-value to measure the confidence of association. Z-score was also obtained to examine the activation or inhibition status of a particular pathway in the present dataset. For the calculation of Z-score, gene expression from Ingenuity knowledge base was compared with gene expression changes observed in the present dataset. For example, if the activation of a particular pathway was associated with upregulation of a gene in a particular canonical pathway in the knowledge base and the present dataset it was assigned a score of 1. However, if the activation of a particular pathway was associated with a change in gene expression which is opposite of the observed change in the present dataset it was assigned a score of -1. Finally, all the genes which belonged to a particular pathway were examined and a total score was assigned.

Upstream regulator analysis was conducted using IPA software, in order to identify the signal transduction regulators that can mimic the observed gene expression changes in the present dataset. The direction of change in the gene expression observed in the experimental samples (relative to a control) was compared for changes in gene expression observed by application of a particular upstream regulator as published in the literature. Each potential upstream regulator was analyzed by using two statistical measures: an overlap p-value and an activation z-score. The overlap p-value was based on significant overlap between dataset genes and known targets regulated by an upstream regulator. The activation z-score was used to infer activation states of upstream regulators based on comparison with a model that assigns random regulatory directions.

#### Validation of upstream regulators

Maternal renal subcellular protein fractions for cytosol and nucleus were obtained using a subcellular protein fractionation kit for tissues (Pierce Thermo Fisher Scientific). Protein lysates were then analyzed by Western immune blotting, as previously described [17]. Briefly, the protein samples (50 *μ*g total extract, 30 *μ*g cytosolic extract or 15 *μ*g nuclear extract) were heat denatured in Laemmli buffer, separated on sodium dodecyl sulfate polyacrylamide gel electrophoresis (SDS-PAGE), and transferred to polyvinylidene fluoride membranes. Membranes were blocked in 5% non-fat dried milk in 0.05% Tris-buffered saline (TBST) for 1 h, and then probed in primary antibody overnight at 4°C. The primary antibodies, dilution factors, and vendor information is shown in S2 Table. After three 10 min washes with TBST, the membranes were incubated with corresponding secondary antibodies that were diluted at 1:1000. Bound antibodies were visualized using the SuperSignal West-Femto or Pico substrate (Pierce ThermoFisher, Pittsburg, PA). Digital images were captured using the Alpha Innotech ChemiImager Imaging System with a high-resolution charge-coupled device camera and quantified using the Alpha Innotech ChemiImager 4400 software (Cell Biosciences, Santa Clara, CA). Relative total protein expression was calculated with respect to β-Actin (ACTB), cytosolic protein expression with respect to heat shock protein 90 (HSP90), and nuclear protein expression with respect to TATA-binding protein (TBP). To compare band densitometries of the same protein between different gels, a standard sample was used in every gel. Results are expressed as folds of CD-NP.

### Statistical analysis

Non-omics data was presented as means ± SE. To determine the significance of rat strain, pregnancy, or rat strain x pregnancy interaction, we used two-way ANOVA, after confirming equal variances via Levene analysis. Differences between rat groups were confirmed using one-way ANOVA of a composite independent variable that combines both rat strain and pregnancy stage and LSD post hoc analysis for parametric data. Mann-Whitney analysis was used for non-parametric data. All statistical analyses were performed using SPSS 26.0 (IBM, Armonk, NY). Statistical significance was determined as *p*<0.05.

## Results

### Renal and cardiovascular adaptation to pregnancy

**Fig 1:**
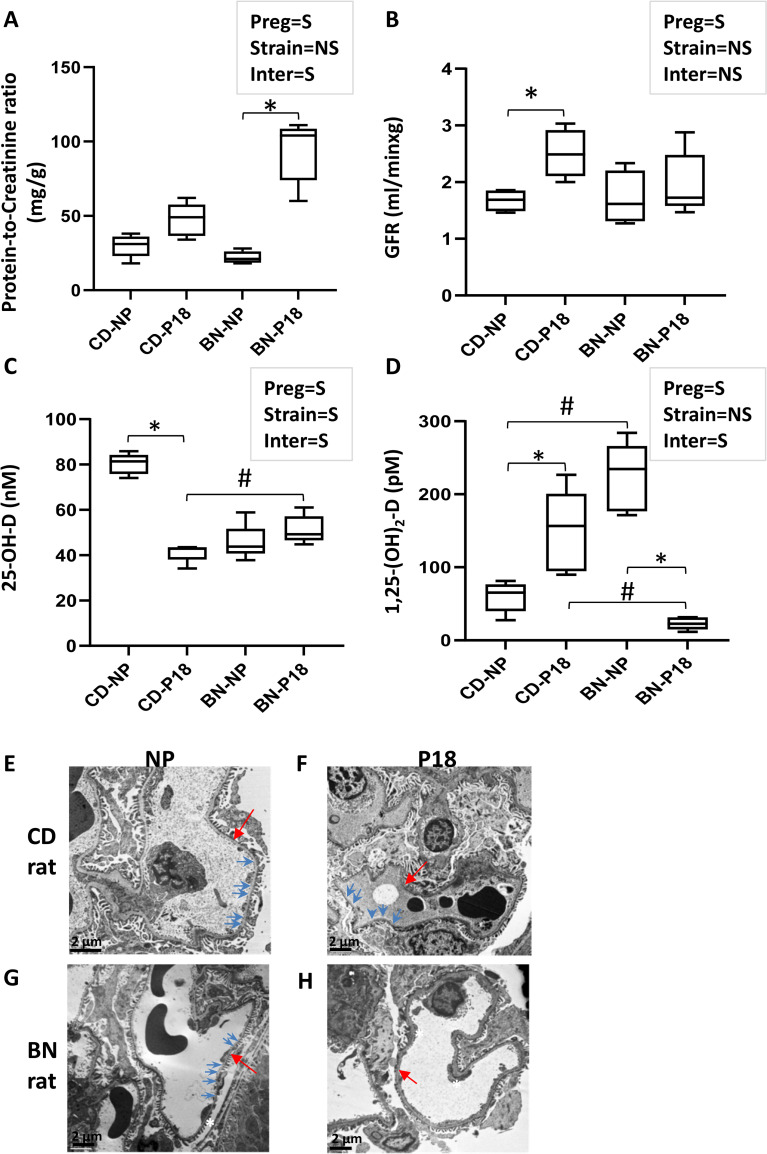
Renal functional and morphometric differences between BN and CD rats: A) Protein-to-Creatinine ratio (mg/g) shows increased proteinuria in pregnant BN rats. B) glomerular filtration rate (GFR; ml/min x g) is increased in pregnant CD rats only. Maternal plasma levels of C) 25-OH-D, and D) 1,25-(OH)_2_-D as determined by ELISA. Bars represent the mean ± SE (n = 5/group). Text boxes show the 2-way ANOVA results for the effect of pregnancy (preg), rat strain (strain) or preg x strain interaction (inter) (S = significant, NS = non-significant). Composite 1-way ANOVA was performed to identify differences among the four groups: *p<0.05 P18 vs NP. ^#^p<0.05 CD vs BN. E-H) Representative electron microscopy photos of glomeruli endothelial cells showed a normal thin basement membrane (red arrow) and multiple fenestrations (blue arrows) in non-pregnant and pregnant CD rats (E, F), and non-pregnant BN(G). Fewer fenestrations and basement membrane thickening of glomerular endothelial cells were observed in pregnant BN renal samples, but without endotheliosis (H).

To study pregnancy-specific renal adaptation we used CD rats that represent healthy pregnancy, and BN rats that model pregnancy complicated by placental insufficiency, fetal growth restriction, and preeclampsia-like symptoms. We found that pregnant BN dams show twice the total protein-to-creatinine ratio compared to age-matched non-pregnant BN rats, while CD rats did not show a significant change in the total proteinuria levels compared to non-pregnancy (Fig 1A). Furthermore, we found that pregnancy induced a 50.3% increase in GFR in CD rats while only increasing GFR by 12.2% in BN rats (Fig 1B, p<0.05). Pregnancy induced striking changes to the plasma levels of vitamin D metabolites. In BN rats, pregnancy induced a 9.9-fold decrease in 1,25-(OH)_2_-D plasma levels compared to non-pregnancy with no major changes in 25-OH-D levels (Fig 1C and 1D). In contrast, in CD rats, pregnancy induced a 2-fold decrease in the plasma levels of 25-OH-D and a 2.5-fold increase in the levels of 1,25-(OH)_2_-D compared to non-pregnancy (Fig 1C and 1D). Electron microscopy studies showed similar glomerular endothelial structures between non-pregnant and pregnant CD rats (Fig 1E and 1F). Pregnant BN rat glomeruli endothelial cells show thicker basement membranes and decrease in fenestrations compared to pregnant CD rats and non-pregnant BN rats (Fig 1H vs 1G or 1F). However, glomerular endotheliosis was not observed in any sample.

In addition, BN rat pregnancies showed smaller fetal and placental weights, and smaller litter sizes than CD rat pregnancies (S2A and S2B Fig). The fetal resorption rate was 2±2% for CD rat litters and 23±6.2% (p<0.05). Furthermore, pregnant BN rats showed significant increases in maternal heart rate and maternal blood pressure compared to non-pregnant BN rats (S2C and S2D Fig). In contrast, pregnant CD rats showed a 10 mm Hg decrease in maternal blood pressure blood pressure and no change in heart rate compared to non-pregnancy (S2C and S2D Fig). Of interest, two-way ANOVA analysis determined that pregnancy but not rat strain had a significant effect on renal parameters such as proteinuria and GFR (Fig 1), while rat strain but not pregnancy had a significant effect on cardiovascular parameters such as HR and MAP (S2 Fig). Importantly, the interaction of pregnancy with rat strain was significant for both maternal renal and cardiovascular parameters.

### Pregnancy and rat strain effects on renal transcriptome and DNA methylome

To uncover potential key molecules involved in the renal adaptations induced by pregnancy in two rat strains with different reproductive phenotypes, we constructed 12 RNA-seq libraries, and 12 RRBS methylation libraries from 12 rats (n = 3 for each group: BN NP, BN P18, CD NP and CD P18). DNA and RNA were extracted from a representative kidney section. High quality reads were obtained from both RNA-seq and RRBS sequencing (S3 Fig). In sum, ~326 million 100-bp single-end RNA-Seq reads and 234 million 75 bp RRBS reads were generated, corresponding to 25 million sequence reads per RNA-Seq sample and 19.6 million sequence reads per RRBS sample (S3 Fig). On average, 82.7% of the RNA-seq reads and 70.1% of RRBS reads were aligned uniquely to the rat genome (S4 Fig and S3 Table). Annotation of aligned reads revealed an average of 15,000 transcripts in transcriptome data and 1.14 million CpG sites covered by at least 10 reads in methylome data.

**Fig 2:**
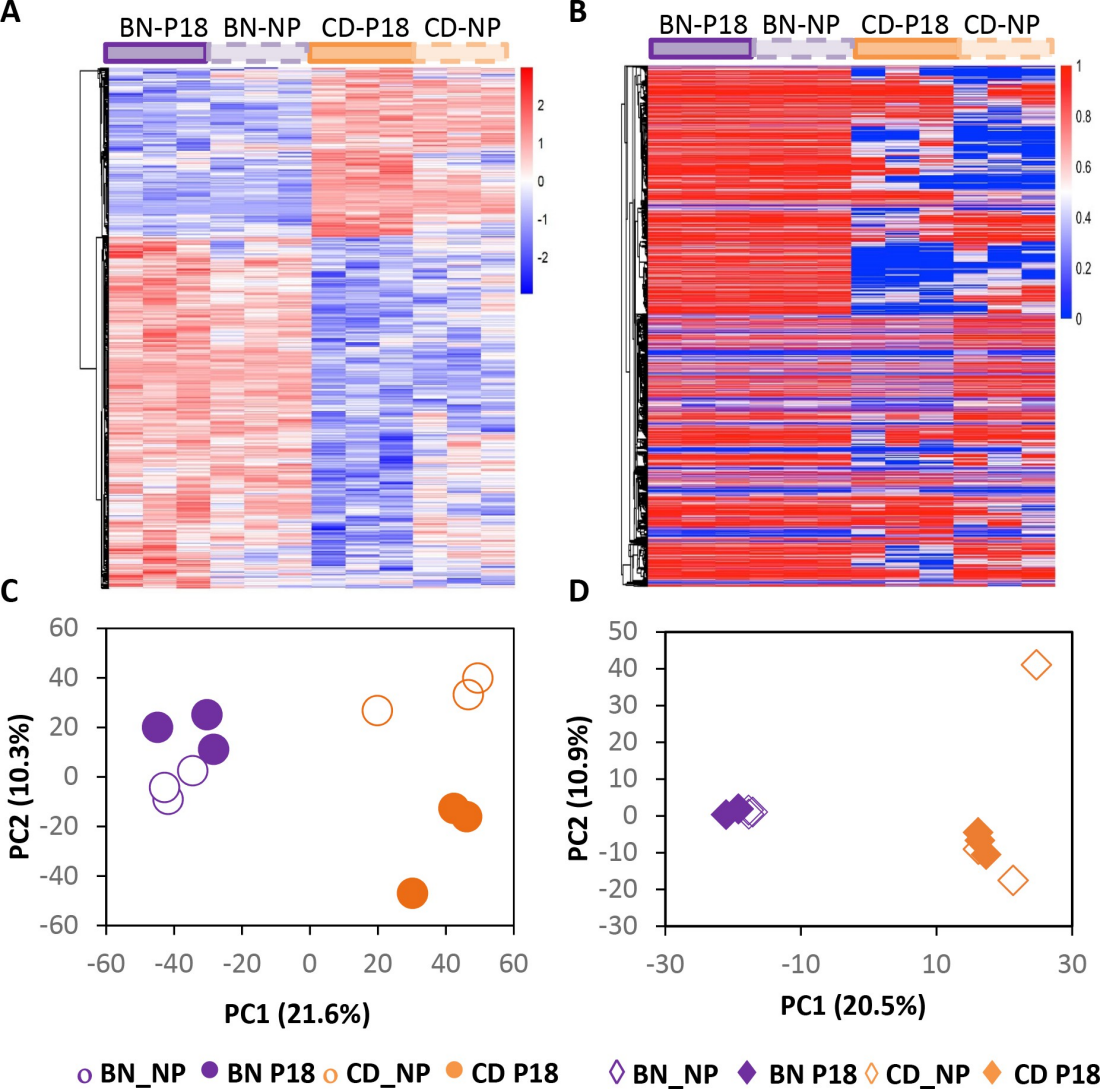
Role of pregnancy and rat strain on renal transcriptomic and methylomic profiles. Heatmap (A) and PCA plot (B) of all DEGs between BN-P and CD-P. Heatmap (C) and PCA plot (D) of all DMRs between the four rat groups. n = 3 for each rat group.

After normalization of sequencing depth, sample correlation was calculated using “Euclidean” method from all differentially expression genes (DEGs) and differentially methylated CpGs (DMCs), using FPKM (Fragments Per Kilobase of transcript per Million mapped reads) and methylation beta values, respectively. Fig 2A and 2B demonstrate the correlation heat maps from both RNA-seq and RRBS data, which show a clear difference between BN and CD rat strains at both transcriptome and methylome levels.

Data also reveal that pregnancy induces significant changes in transcriptome and methylome and is the second most important factor distinguishing the samples. Further, our data reveals rat strain is the major factor in determining transcriptome and methylome differences between BN and CD rats, as determined by the Principal Component Analysis (PCA) (Fig 2C and 2D). Data also shows that pregnancy induces more remarkable change in transcription than in methylation, and the CD rat’s response to pregnancy is more noticeable than that of BN rats (Fig 2C and 2D).

### Pregnancy-induced gene expression changes

**Fig 3:**
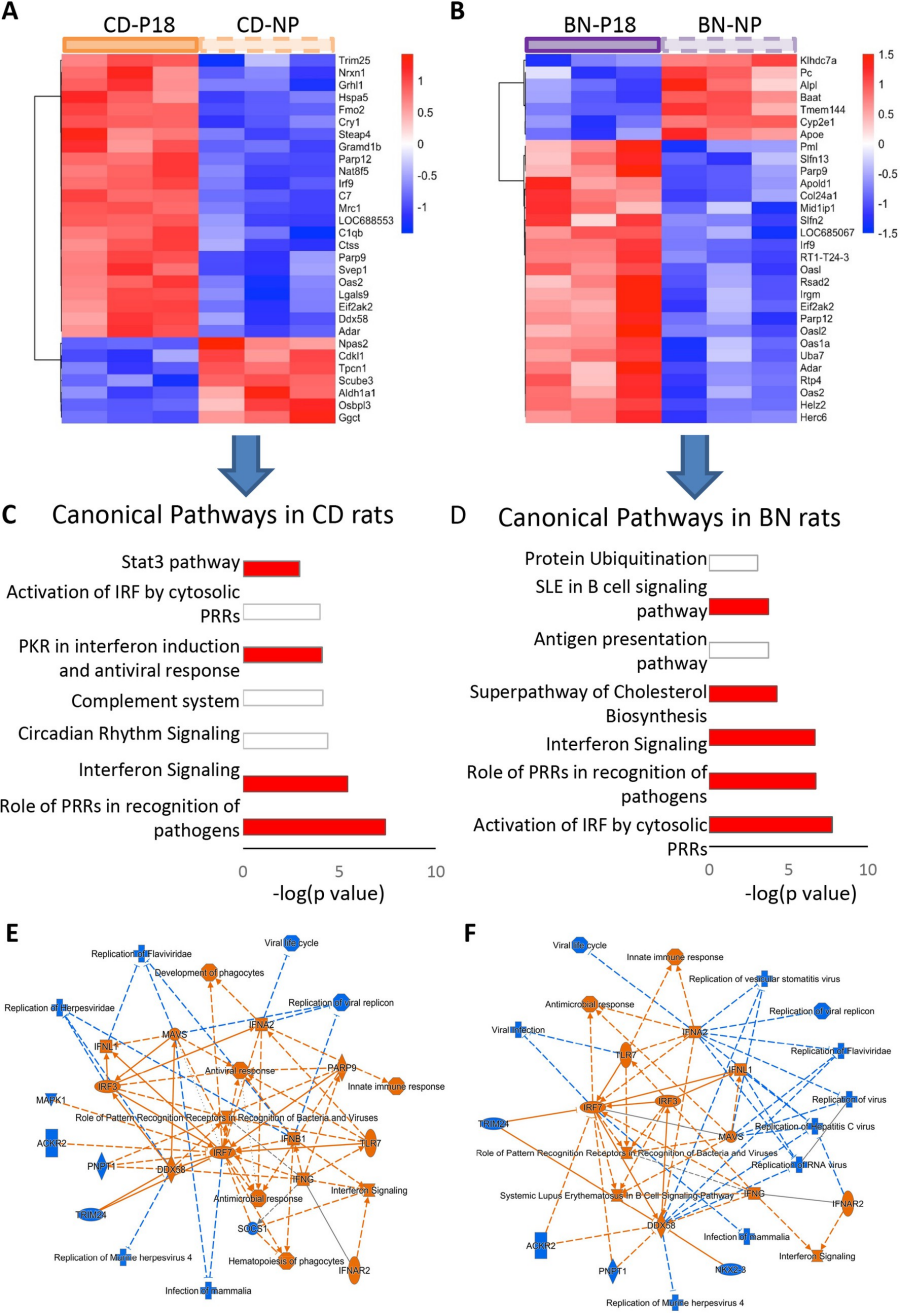
Effect of pregnancy on renal transcriptome. Heatmaps show the top 30 pregnancy-induced DEG in CD (A) and BN (B) rats. Pathway analysis determined the top canonical pathways altered by pregnancy in CD (C) and BN (D) rats. (red: activation) Summary of the effect of pregnancy on transcriptomics pathways in CD (E) and BN (F) rats (orange: activation, blue: inhibition).

By comparison of renal transcriptomic profiles in pregnant and non-pregnant rats, we identified 297 DEGs in CD rats and 174 DEGs in BN rats (S4 Table). Of those, pregnancy induced similar expression changes (and in the same direction) in 60 genes. However, there were 351 pregnancy induced DEGs specific to one rat strain (237 DEGs specific to CD rats and 114 DEGs specific to BN rats), indicating differential molecular adaptation to pregnancy according to rat strain. Heat maps of the top 30 DEGs show differential gene sets with only 5 shared DEGs (Irf9, Parp9, Oas2, Eif2ak2, and Adar) (Fig 3A and 3B).

However, Ingenuity pathway analysis indicated similar activation of innate immune pathways such as the ‘Role of Pattern Recognition Receptors’ and ‘Interferon signaling’ in both CD and BN rat strains (Fig 3C and 3D). Moreover, similar upstream regulators are involved in the pregnancy-induced temporal transcriptomic changes in both BN and CD rat kidneys, with interferon regulatory factor 7 (IRF7) as a key upstream regulator that is predicted to be activated by pregnancy in both rat strains (Fig 3E and 3F, and S5 Table). Immunoblotting confirmed that pregnancy upregulated maternal renal IRF7 protein levels in both rat strains (S5 Fig). The principal pathways in both CD and BN rats show activation of innate immune pathways to inhibit viral and microbial replication. However, BN rat kidneys also show pregnancy-induced activation of necroptosis signaling pathways, superpathway of cholesterol synthesis, and the systemic lupus erythematosus in B cell signaling pathway (Fig 3D and 3F, and S5 Table).

### Rat strain-dependent differential gene expression

**Fig 4:**
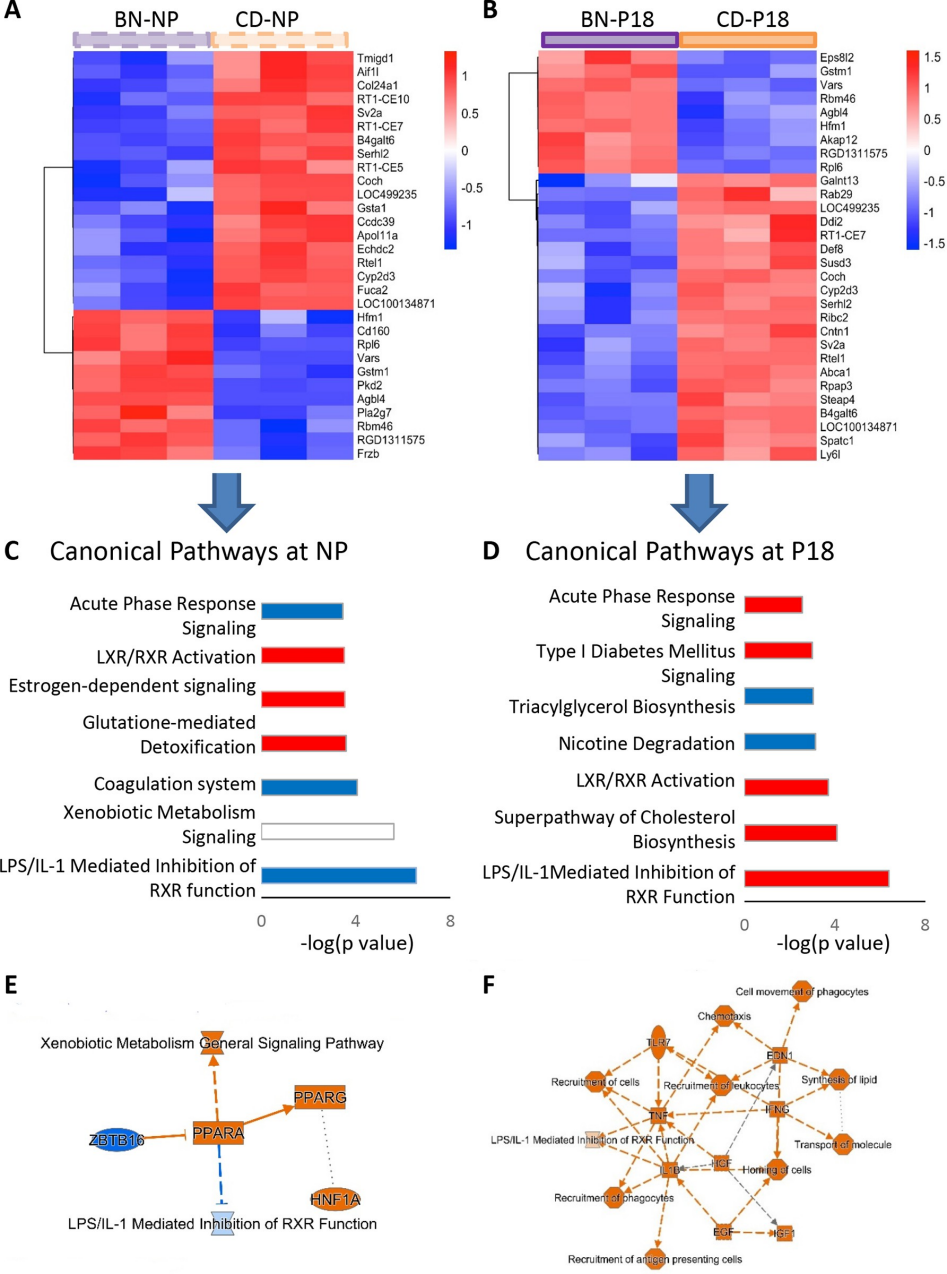
Effect of rat strain on renal transcriptome. Heatmaps show the top 30 rat-strain dependent DEGs at non-pregnancy (A) and at pregnancy day 18 (B) rats. Pathway analysis determined the top canonical pathways that are different in BN compared to CD at non-pregnancy (C) and at pregnancy day 18 (D) (red: activation, blue: inhibition). Summary of the rat strain (BN vs. CD) differences in transcriptomics pathways at non-pregnancy (E) and at pregnancy (F) (orange: activation, blue: inhibition).

Comparison of BN with CD rat renal transcriptomic profiles at either non-pregnancy or pregnancy, revealed 569 DEGs at P18 and 475 DEGs at NP (S4 Table). At non-pregnancy, there were 198 DEGs that were downregulated and 277 DEGs that were upregulated in BN compared with CD. At pregnancy day 18, there were 185 DEGs that were downregulated and 384 DEGs that were upregulated in BN compared with CD. There were 205 DEGs in both non-pregnancy and pregnancy datasets. Of interest, 23 of these showed differential regulation at pregnancy (S4 Table) that in addition to the 364 DEG at pregnancy add to 387 DEGs affected by both rat strain and pregnancy. Altogether, only 36% of the DEGs at P18 were strictly aligned with rat strain (i.e. genetics). This is shown in heatmaps of the top 30 DEGs (Fig 4A and 4B), where 30% of DEGs were shared (*RT1-CE7*, *LOC499235*, *LOC100134871*, *RGD 1311575*, *Vars*, *Cyp2d3*, *B4galt6*, *Agbl4*, *Hlfm1*). IPA of all DEGs identified different canonical pathways at non-pregnancy compared to pregnancy (Fig 4C and 4D, and S6 Table). For instance, the LPS/IL-1 inhibition of RXR function and the acute phase response pathways were decreased at non-pregnancy but activated at pregnancy in BN compared to CD. The coagulation system was inhibited at non-pregnancy but not at pregnancy in BN compared to CD. Additional protective systems such as activation of glutathione-detoxification pathway and inhibition of HIF1α signaling were evident at non-pregnancy but not at pregnancy in BN compared to CD (Fig 4C and 4D and S5 Table). In addition, activation of cholesterol biosynthesis superpathway and production of nitric oxide and reactive oxygen species were only shown at pregnancy.

**Fig 5:**
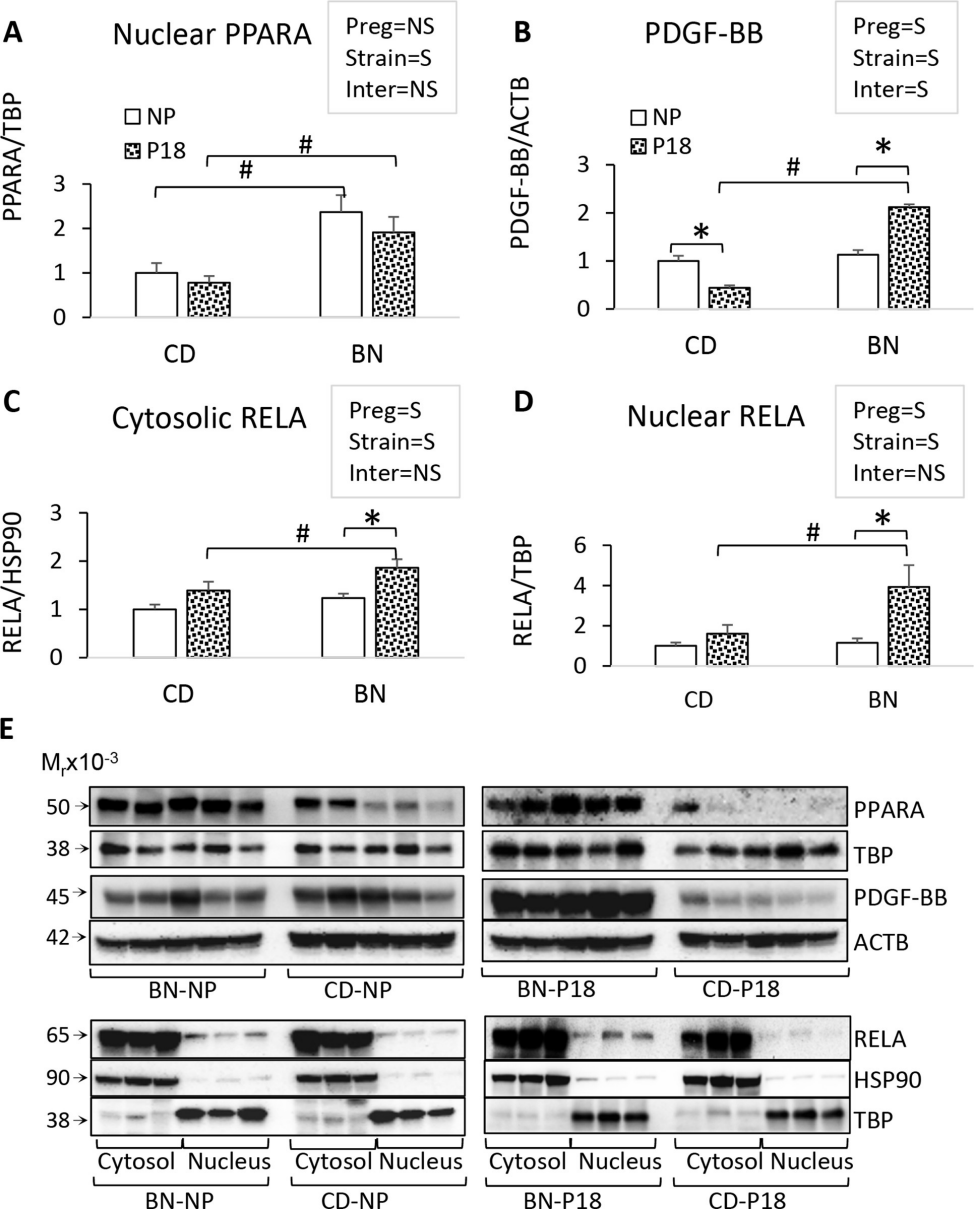
Key upstream regulators of rat-strain DEGs. Rat kidney protein fractions were studied by SDS-PAGE and immunoblotting for relative PPARA to TBP levels in nuclear protein extracts (A); relative PDGF-BB dimer to ACTB protein levels in whole protein extracts (B); relative cytosolic NF-kB p65 (RELA) to HSP90 protein levels (C) and nuclear RELA to TBP protein levels (D). E) Representative immunoblots are shown in G. Bars represent the average ± error, n = 5 rats/group. Text boxes show the 2-way ANOVA results for the effect of pregnancy (preg), rat strain (strain) or preg x strain interaction (inter) (S = significant, NS = non-significant). Composite 1-way ANOVA was performed to identify differences among the four groups for RNA-seq validation. *p<0.05 P18 vs NP, ^#^p<0.05 BN vs CD.

Other canonical pathways were similarly affected by rat strain at non-pregnancy and pregnancy, including LXR/RXR pathway, xenobiotic metabolism, nicotine metabolism, and estrogen biosynthesis. The main gene pathways between BN and CD rats at non-pregnancy (Fig 4E) and pregnancy (Fig 4F) are drastically altered by pregnancy: BN show increased anti-inflammatory and xenobiotic metabolism at non-pregnancy, with a shift towards inflammatory, growth and repair pathways at pregnancy in comparison with CD rats (Fig 4E and 4F). At non-pregnancy, the top upstream regulators that explain the differences between BN and CD rats was peroxisome-proliferator-activated receptor alpha (PPARA) (Fig 4E, S6 Table). In contrast, at P18, the top predicted upstream regulators were platelet derived growth factor-B homodimer (PDGF-BB), and pro-inflammatory mediators such as tumor necrosis factor (TNF), all with predicted activation (S6 Table). Immunoblotting confirmed that BN rats express significantly higher levels of PPARA not only at non-pregnancy but at pregnancy stages (Fig 5A). Furthermore, we show that the upstream regulator PDGF-BB was differentially regulated by pregnancy according to rat strain, with pregnancy increasing PDGF-BB in BN rats and decreasing it in CD rats (Fig 5B).

Because multiple pro-inflammatory mediators were predicted upstream regulators of BN/CD differences at pregnancy, we investigated the activation of the NF-κB p65 (RELA) transcription factors. Inactive RELA molecules reside in the cytosol and are translocated to the nucleus upon activation. We studied the levels of both cytosolic and nuclear RELA and found there were no differences in renal cytosolic or nuclear RELA protein levels between BN and CD rat at non-pregnancy (Fig 5C and 5D) but BN pregnant rats had significantly higher renal cytosolic and nuclear RELA protein levels compared to CD pregnant rats (Fig 5C and 5D). Importantly, pregnancy induced a significant increase in the levels of nuclear RELA (activated) only in BN rats (Fig 5D). These data confirm that both PPARA, PDGF-BB, and NF-κB are upstream regulators of the observed differences between rat strains, especially during pregnancy.

### Validation of RNA-seq data

**Fig 6:**
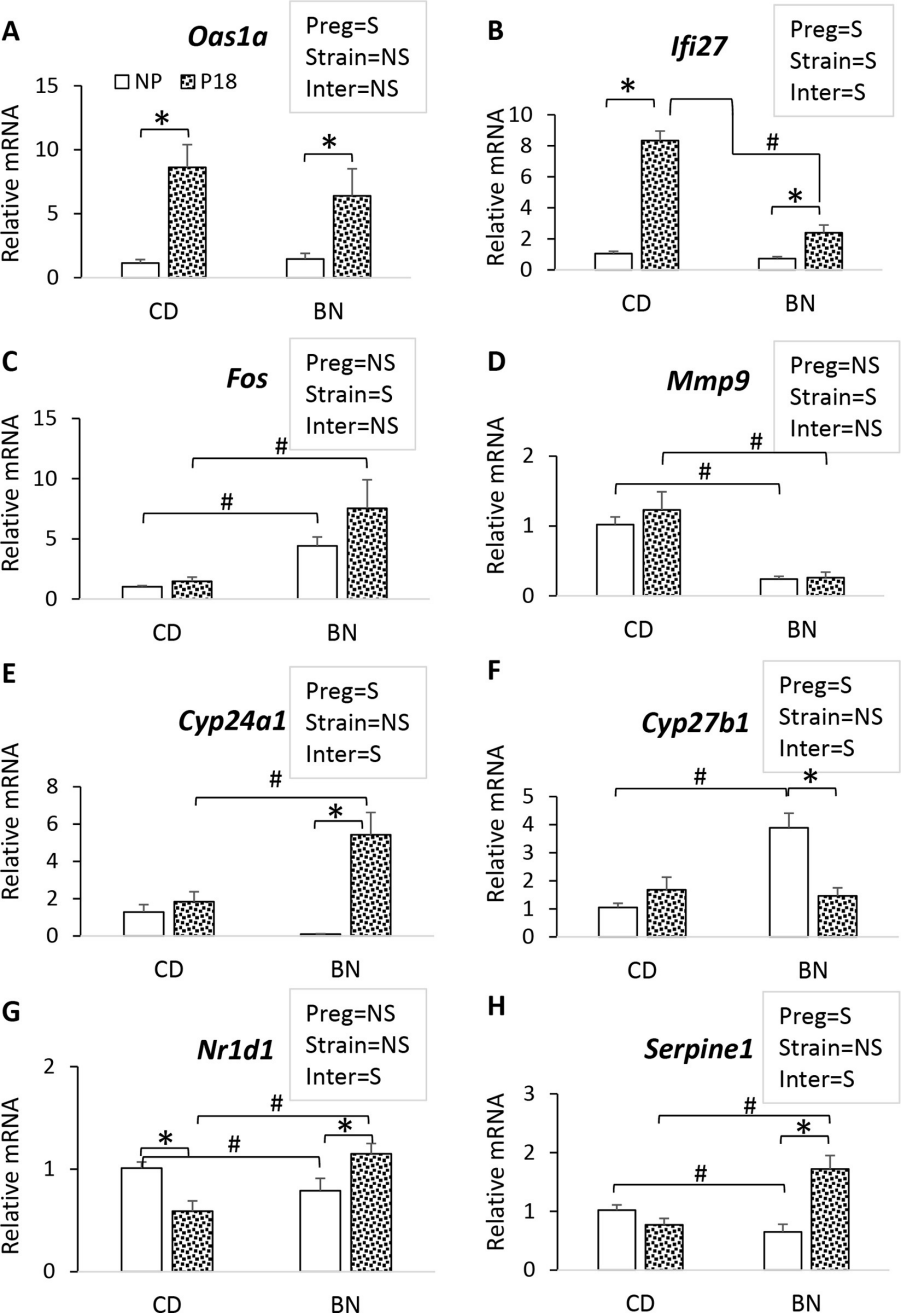
Validation of RNA-sequencing data. Quantitative SYBR green PCR was used to study 8 genes selected to validate DEGs from all four comparisons (BN_P/BN_NP, CD_P/CD_NP, BN_NP/CD_NP, and BN_P/CD_P). Two genes were selected on the basis of pregnancy regulation: A) *Oas1a*, and B) *Ifi27*. Two genes were selected based on rat strain differences, C) *Fos*, D) *Mmp9*, and 4 genes on differential regulation by pregnancy according to rat strain: E) *Cyp24a1*, F) *Cyp27b1*, G) *Nr1d1*, and H) *Serpine1*. mRNA levels are shown as fold of CD NP values. Bars represent the average ± error, n = 5 rats/group. Text boxes show the 2-way ANOVA results for the effect of pregnancy (preg), rat strain (strain) or preg x strain interaction (inter) (S = significant, NS = non-significant). Composite 1-way ANOVA was performed to identify differences among the four groups for RNA-seq validation. *p<0.05 P18 vs NP, ^#^p<0.05 BN vs CD.

Transcriptomic datasets were validated in selected DEGs (Fig 6) using the same 12 kidney samples plus additional 8 kidneys to cover the entire rat cohort A (S1 Fig). 2’5’-Oligoadenylate synthase 1A (*Oas1a*) and interferon alpha inducible protein 27 (*Ifi27*), two genes that participate in innate immune pathways, were significantly upregulated by pregnancy in both rat strains (Fig 6A and 6B).

Furthermore, pregnancy-induced upregulation of *Oas1a* is similar in both rat strains while upregulation of *Ifi27* is significantly higher in pregnant CD rats compared to pregnant BN rats, once again validating the RNA sequencing data. We also selected two DEGs that are rat-strain dependent, the pro-inflammatory transcription factor *Fos* and the matrix metalloproteinase *Mmp9*. *Fos* is significantly lower in CD rats compared to BN rats (Fig 6C) and *Mmp9* is significantly higher in CD rats compared to BN rats (Fig 6D), once again confirming RNA sequencing datasets (S3 Table). Finally, we investigated four DEGs with opposite directionality of regulation between non-pregnancy and pregnancy, those involved in 1, 25-(OH)_2_-D synthesis (*Cyp27b1*)/catabolism (*Cyp24a1*) (Fig 6E and 6F), that of the orphan nuclear receptor Rev-ErbA or *Nr1d1* (Fig 6G), and of plasminogen activatory inhibitor 1 or *Serpine1* (Fig 6H). The upregulation of the vitamin D 24-hydroxylase *Cyp24a1* and downregulation of vitamin D 1α-hydroxylase *Cyp27b1* in BN pregnancy, but not in CD pregnancy, was confirmed (Fig 6E and 6F). Of note, renal changes in *Cyp24a1* and *Cyp27b1* mRNA levels suffice to explain plasma 1,25-(OH)_2_-D levels. For both *Nr1d1* and *Serpine1*, pregnancy induced an opposite effect on CD compared to BN, i.e., expression was downregulated in pregnant CD, but upregulated in pregnant BN. Altogether, the expression fold differences and directionality of expression differences shown in RNA sequencing datasets were confirmed.

### DEGs associated with altered DNA methylation

**Fig 7:**
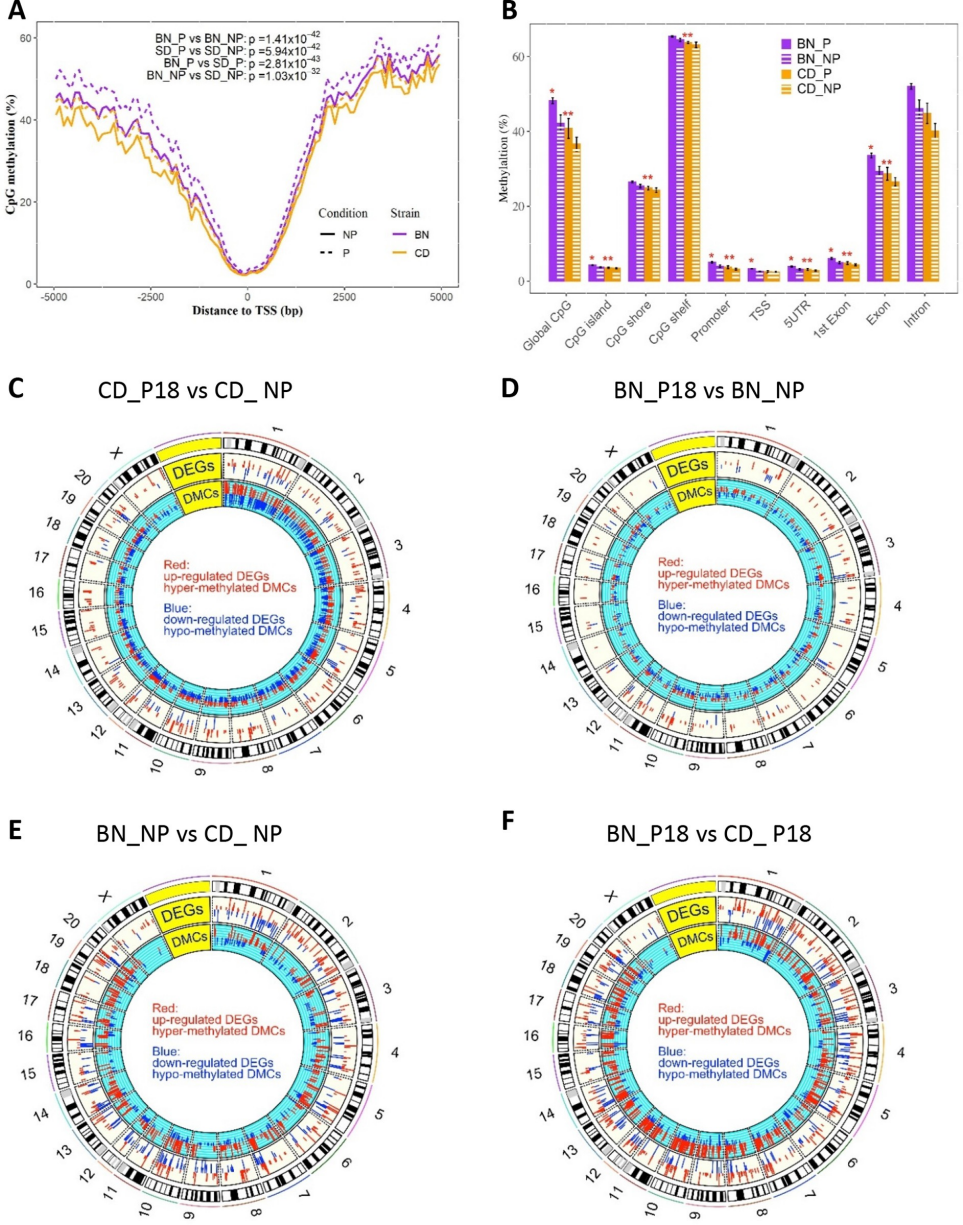
Genome-wide DMRs and integration with DEGs. A) CpG methylation levels in relation to TSS, B) Average CpG methylation levels according to genomic features. Integration of renal DMRs and DEGs is shown in circus plots of: C) pregnant CD compared to non-pregnant CD, D) pregnant BN versus non-pregnant BN, E) non-pregnant BN compared to non-pregnant CD, and F) pregnant BN versus pregnant CD. Lines/bars represent the average ± error (n = 3/group, 12 total). Paired t-test and two-tailed t-test was used to determine the significance in (A) and (B), respectively. *p<0.05 BN_P vs BN_NP, **p<0.05 BN_P vs CD_P.

To identify the role of DNA methylation on the temporal transcriptomic changes, we investigated the genome distribution of CpG sites in all four groups (Fig 7).

In all groups, the CpG methylation was low at TSS and its close proximity, but was significantly different at CpGs 500 bp away in both directions (Fig 7A). However, there were no significant differences in methylation at various genomic features between CD_NP compared to CD_P18 or BN_NP compared to CD_NP (Fig 7B). Pregnant BN showed significantly higher methylation than non-pregnant BN of CpG island, promoter, TSS, 5-UTR, 1^st^ exon, and other exon regions (Fig 7B and *p<0.05 BN_P vs BN_NP). In addition, pregnant BN also demonstrated higher methylation than pregnant CD at similar structure, as well as CpG shelf regions (Fig 7B and **p<0.05 BN_P vs CD_P).

Integration of DMRs and DEGs is illustrated in circos plots (Fig 7C–7F) and the correlation between DMR and regulation directions of DEGs is shown in S6 Table. Pregnancy only induced 7 overlapping DMR/DEGs in CD rats (Fig 7C), and 6 overlapping DMR/DEGs in BN rats (Fig 7D); none of the DMRs were in the promoters, and only 2 DMRs in CD rats showed a reverse correlation with the DEGs (S7 Table). In contrast, comparison of rat strains resulted in 65 overlapping DMRs with 35 DEGs, of which only 28 DMRs showed a reverse correlation with the corresponding 13 DEGs in non-pregnant BN compared to non-pregnant CD rats (Fig 7E and S7 Table). There were 89 overlapping DMRs with 46 DEGs, of which only 36 DMRs had a reverse correlation with respect to the 21 corresponding DEGs (Fig 7F, S7 Table). Furthermore, only one DEG showed overlapping DMRs in the promoter region, that of metallothionein 1 gene (*Mt1*). Altogether, pregnancy-dependent and rat-strain-dependent DEGs did not correlate with changes in promoter methylations.

**Fig 8:**
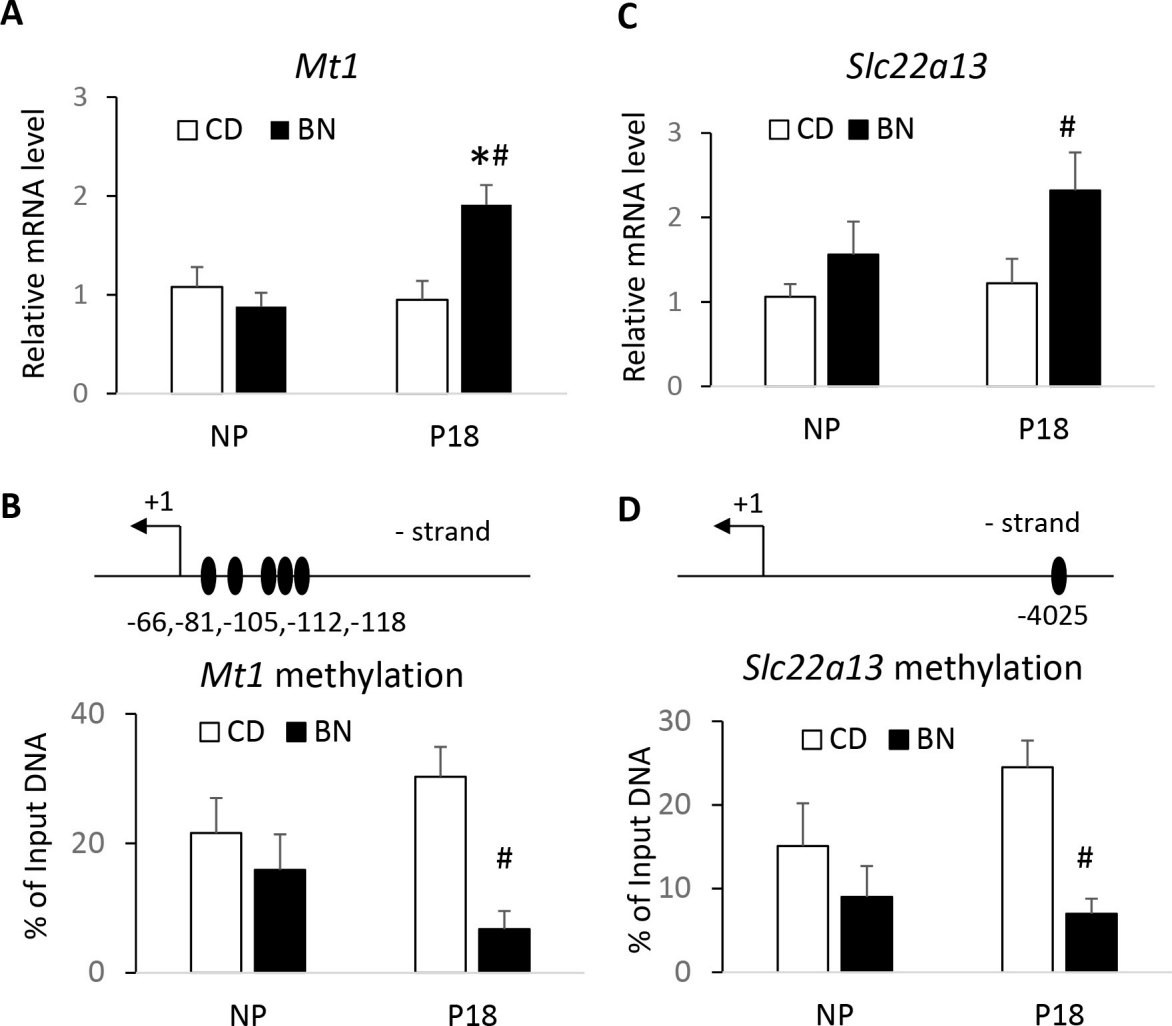
Validation of integration analysis of DEGs/DMRs. DEGs were validated by real-time PCR and DMRs by MeDIP. mRNA abundance (A) and promoter methylation (B) of metallothionein 1 (*Mt1*). mRNA abundance (C) and 1-5kb methylation (D) of solute carrier organic cationic transporter family 22 member 13 (*Slc22a13*). mRNA levels are shown as fold of CD NP values, and % methylation is calculated as % of input gDNA levels. The number and position, relative to the TSS, of the CpGs studied is also shown. Bars represent the average ± error, n = 5 rats/group. *p<0.05 P18 vs NP, ^#^p<0.05 BN vs CD.

We validated the integration analysis by studying two genes with potential impact on kidney metabolism/function. First, we investigated the expression (Fig 8A) and promoter methylation (Fig 8B) of metallothionein 1 (*Mt1*), the only gene that showed overlapping promoter DMRs/DEG in opposite direction.

Our omics analysis showed a 2.1-fold upregulation of *Mt1* mRNA and hypomethylation at 4 promoter CpGs (~30% difference), and 1 CpG at the 5’UTR (~12% difference) in pregnant BN rats compared to pregnant CD rats (S3 Table). Real-time PCR confirmed a significant 2.1-fold upregulation of *Mt1* expression in pregnant BN over pregnant CD (Fig 8A). MeDIP of a 120 bp promoter region containing 5 DMRs showed that pregnant CD rats had a 23.6% higher methylation of that region compared to pregnant BN rats. Of interest, real-time PCR demonstrated a significant upregulation of *Mt1* in pregnant BN in comparison with non-pregnant BN (Fig 8A) absent from the RNA-seq analysis. Second, we studied the mRNA expression and methylation levels at 1-5kb region of *Slc22a13*, an organic anion/urate transporter belonging to the solute carrier family. Omics analysis showed a 2.6-fold upregulation of *Slc22a13* mRNA and hypomethylation of 26.6% at a single CpG site at -1583 of the TSS (1–5 kb region) in pregnant BN rats compared to pregnant CD rats (S3 Table). We found a 2.04 fold increased expression and a 17.5% hypomethylation of a region containing this CpG in pregnant BN compared to pregnant CD rats (Fig 8C and 8D).

## Discussion

We are reporting renal maladaptation to pregnancy induced physiological and molecular changes in BN rats. Previous reports have shown that BN rats are a useful model of placental insufficiency caused by shallow trophoblast invasion and remodeling of uterine arteries [27–29]. Placental complications include reduced uteroplacental blood flow with increased angiogenesis; associated with fetal growth restriction and fetal resorption that is worse in early to mid-gestation with subsequent catch-up growth in the last days of gestation [15, 16, 27]. Longitudinal studies in BN rat pregnancies also showed mild-features of preeclampsia; we reported a rise in maternal blood pressure in day 14 of gestation with a 45 mm Hg increase by day 17, together with mild proteinuria [15]. An interesting feature of BN pregnancies is maternal 1,25-(OH)_2_-D deficiency [16] that has also been reported in preeclampsia [11, 17]. Although it is thought that the cause of vitamin D deficiency is derived from a defective placenta, dysregulation of vitamin D metabolism has been observed at pre-pregnancy and postpartum stages in women that developed preeclampsia [17]. In this study we found similar mild preeclampsia-like symptoms and confirmed maternal 1,25-(OH)_2_-D deficiency in BN pregnancies, albeit glomerular endotheliosis, a PE renal hallmark, was absent.

### Pregnancy-induced changes in renal function

Pregnancy is thought to increase GFR via cardiometabolic adaptations which include increased cardiac output and volume [30, 31]. Furthermore, the hormonal and growth factor milieu induce kidney hyperplasia which leads to a drastic increase in kidney size of about 25% [30, 31]. In accordance with these data, we found that the widely used CD rat model of healthy pregnancy showed a significant pregnancy-induced increase in GFR. In contrast, BN rat pregnancies showed a blunted increase in GFR and significant albeit mild increase in total protein-to-creatinine ratio. However, morphological studies did not reveal glomerular endotheliosis, a pathognomonic feature of preeclampsia. Some established models of preeclampsia like the reduced uterine perfusion pressure (RUPP) model and the transgenic renin-angiotensinogen rat model also lack glomerular endotheliosis [32–34], while the soluble Flt1 model of PE does have this feature, making the latter model more suitable to investigate renal damage induced by PE [34–36]. Another interesting finding was that pregnancy alone, but not rat strain, was a significant regulating factor in the renal parameters (Fig 1A and 1B) while rat strain alone and not pregnancy was significantly associated with cardiovascular parameters (S2C and S2D Fig). This suggests that the basal renal function of BN and CD rats did not differ at non-pregnancy and a similar direction of change was induced by pregnancy in both rat strains. In contrast, pregnancy induced a different adaptation in both heart rate and blood pressure with BN rats showing a significant pregnancy-increase in both parameters. Altogether, these findings suggest that the genetic/epigenetic background of BN and CD rats plays a stronger role in the maternal cardiovascular adaptation compared to the renal adaptation to pregnancy.

### Pregnancy-induced transcriptomic changes

Pregnancy-specific temporary changes in renal structure are likely to be preceded by changes in gene-pathway networks, induced by placental derived hormones and growth factors [37]. However, currently there is no data on renal transcriptomics and methylome changes in healthy or complicated pregnancies, either humans or animals. Therefore, this study is the first to uncover renal transcriptome and methylome changes in rat models of healthy and complicated pregnancies. We have found that pregnancy induced more transcriptomic changes in CD rats compared to BN rats, suggesting that BN rats show some level of resistance to pregnancy-induced stimuli. There were 60 DEGs that were common between CD and BN pregnant rats, representing only 20.2% and 34.4% of all DEGs induced by pregnancy in CD and BN rats, respectively. Of interest, integration of methylome with transcriptome data revealed that pregnancy-induced DEGs were not mediated via promoter methylation, thereby suggesting that pregnancy alters renal transcription via short-term transcriptional/translational and posttranslational mechanisms. The lower transcriptomic changes induced by pregnancy in BN rats could be due to placental insufficiency in the production/release of specific factors, intrarenal resistance to placental factors, or both. Previous studies have found progesterone receptor resistance in uterine tissue of BN rats [28], therefore it is possible that nuclear receptors, such as the estrogen receptor, show decreased activation leading to a smaller adaptation towards the same signal. Nevertheless, pathway analysis revealed similar canonical pathways induced by pregnancy in both rat strains, in particular antiviral and innate immune activation pathways, suggesting similarities between rat strains on pregnancy-regulation of key gene pathways. Of interest, there were similar activation of innate immune gene networks by pregnancy in FV1 mice [38], suggesting genetic conservation in molecular mechanisms of adaptation to pregnancy across mammalian species.

### Rat-strain dependent transcriptomic alteration

There is an important genetic component involved in various pregnancy complications. In this study, transcriptomic profiles revealed a higher number of rat strain-dependent DEGs than pregnancy-dependent DEGs that suggest an important role of the epigenetic/genetic background of each rat strain in regulation of renal genes. Of interest, there were many DMRs between rat strains, but integration of DEGs and DMRs revealed that DMRs were not positioned in promoters or CpG islands. Therefore, rat strain-dependent DEGs are likely a result of CpGs on non-promoter regions together with rat-strain specific genetic polymorphisms. Furthermore, there were more DEGs between rat strains when there were compared at pregnancy stages. These data suggest that the genetic/epigenetic background interacts with the pregnancy environment. This was clearly seen by the differential activation/inhibition of canonical pathways. For instance, at non-pregnancy, BN kidney transcriptomic profiles showed inhibition of the ‘acute phase reaction’, ‘LPS/IL1 inhibition of RXR function’, and ‘coagulation pathways’, compared to non-pregnant CD rat kidneys. In contrast, at pregnancy, there was activation of the former 2 inflammatory pathways in BN compared with CD rats. Altogether, rat strain specific DEGs could participate in the predisposition of BN towards renal mal-adaptation to pregnancy. For example, the pro-inflammatory transcription factors *Fos*, *FosB*, and *JunB* were upregulated, while pro-remodelling factors such as *Mmp9* were downregulated in kidneys of pregnant BN rats compared with pregnant CD rats. *Fos* is an important upstream regulator implicated in human adult kidney disease as well as in animal models of glomerulonephritis [39, 40]. Other novel findings are related to metabolic gene differences between rat strains, where the superpathway of cholesterol biosynthesis, the LXR/RXR activation, and glutathione detoxification were upregulated in BN compared to CD rats. CD rats, on the other hand show significantly higher detoxification pathways for lipid peroxides and fatty acid metabolism. We confirmed that BN rat kidneys express higher levels of PPARA, a master upstream regulator of lipid/cholesterol metabolism, cell proliferation, angiogenesis, and inflammation [41], than CD rat kidneys at both non-pregnancy and pregnancy stages. Increased renal nuclear PPARA in BN rats could play a protective mechanism against metabolic stress during pregnancy. Furthermore, PPARs have shown an important role in the maternal adaptation to pregnancy [42] although most studies have been centered on utero-placental PPAR with no studies on maternal renal PPAR. Future research on metabolism of major pathways such as fatty acids and cholesterol in the development of organ damage is therefore warranted.

### Renal transcriptome was regulated by rat strain x pregnancy

This is the first study to show that CD rats present renal adaptations that include negative feedback mechanisms on pro-inflammatory transcription factor RELA as well as on renal PDGF-BB. In contrast BN rats’ adaptation to pregnancy includes activation of RELA and PDGF-BB. PDGF-BB has multiple roles in health and disease [43], in the kidney is a key mediator of mesangial cell proliferation and vascular repair, and plasma PDGF-BB levels are increased in adult renal and cardiovascular diseases [43, 44]. Furthermore, increased maternal plasma PDGF-BB has been observed in pregnancy-induced hypertension and preeclampsia [45] and increased uteroplacental PDGF-BB has been proposed to have a role in defective spiral artery remodeling [46]. Therefore, increased renal PDGF-BB is likely to contribute to the transcriptional and functional changes induced by pregnancy in the BN rat. In addition, the activation of renal RELA in BN pregnancies is in accordance with our previous study showing increased maternal plasma levels of interleukin 6, interferon gamma, and immunoglobulin G in pregnancy compared to non-pregnancy [26], and confirms the canonical pathway activation of ‘acute phase reaction’ in BN rat pregnancies. Of interest, although maternal plasma levels of pro-inflammatory mediators (such as interleukins and TNFs) are elevated in multiple pregnancy disorders [47], very little is known on the maternal organ-specific response to these circulatory factors. This study in conjunction with our previous study on plasma proteomic changes in BN rat pregnancies [26] suggests that the maternal kidney responds to plasma pro-inflammatory cytokines with increases in both NF-kB and AP-1 pro-inflammatory transcription factor activation with subsequent changes in renal transcriptome.

Lastly, pregnancy had a striking interaction with rat strain in terms of vitamin D metabolism. BN rats show lower levels of 25-OH-D but higher levels of 1, 25-(OH)_2_-D at non-pregnancy, with a drastic pregnancy-induced switch towards a 10-fold decrease in maternal plasma 1, 25-(OH)_2_-D levels compared to CD rats. We have observed these features in various different rat cohorts [15, 16]. In addition, we have shown similar pregnancy-induced increases in maternal plasma 1, 25-(OH)_2_-D levels in other species such as sheep and baboon [48, 49]. Pregnant women also experience a significant 2-3-fold increase in maternal plasma 1, 25-(OH)_2_-D levels while women with preeclampsia show smaller increases of this metabolite [10–12, 17]. Although it is thought that vitamin D metabolic dysregulation in preeclampsia is the result of a defective placenta, it is likely that maternal renal dysregulation has an important role in maternal vitamin D metabolic dysregulation. In this study we are showing decreases in maternal plasma 1, 25-(OH)_2_-D levels in the BN rat are due to renal downregulation of *Cyp27b1* (final activation step) and upregulation of *Cyp24a1* (main inactivating step). In contrast, the renal expression of these metabolic enzymes is not affected by pregnancy in CD rats, highly suggesting that increased maternal plasma 1, 25-(OH)_2_-D levels in CD rats is not due to increase renal biosynthesis, but due to an additive effect of placental biosynthesis of 1, 25-(OH)_2_-D. In contrast, this study has shown that in BN rat pregnancies, renal maladaptation is sufficient to explained the drastic deficiency of 1, 25-(OH)_2_-D. Based on various studies, we hypothesize that in CD rat pregnancies, placenta-derived factors induce renal adaptations that include VDR resistance to 1, 25-(OH)_2_-D activation, leading to unaltered levels of *Cyp24a1* and *Cyp27b1*. This VDR resistance can be caused by pregnancy-induced proteasome-degradation of VDR, and would prevent the maternal kidney from sequestering the circulating 1, 25-(OH)_2_-D so that it would be available to the uterus, placenta and fetus. In contrast, we propose that in BN rats, placental insufficiency releases additional pro-inflammatory factors that activate renal pro-inflammatory gene pathways; leading to intrarenal use of plasma 1, 25-(OH)_2_-D to counteract these effects and maintain renal homeostasis. This leads to VDR-dependent upregulation of *Cyp24a1* and downregulation of *Cyp27b1*. One caveat of the increased intrarenal usage of 1, 25-(OH)_2_-D in BN pregnancies is that the current transcriptomic profile is missing multiple vitamin D-sensitive DEGs as shown by transcriptomic studies of 1, 25-(OH)_2_-D-treated non-pregnant animals [50, 51]. Although discrepancies between these transcriptomic profiles derived from non-pregnant animal models could be due to the presence of pregnancy-specific growth factors and hormones, VDR ChIP studies are required to confirm this hypothesis. In sum, we believe these data highly suggest that renal maladaptation to pregnancy could contribute to metabolic dysregulation and vitamin D deficiency observed in pregnancy complications such as preeclampsia.

## Conclusion

This study has several strengths that include parallel studies of transcriptomics with methylomics, functional and morphometric studies of glomeruli, and analysis of circulating vitamin D levels. However, limitations include the estimation of GFR in unconscious rats, the study of pregnancy induced omics and renal function assays using different rat cohorts, and the use of whole kidney sections instead of glomeruli for omics studies. The lack of glomerular endotheliosis in pregnant BN rat kidneys limits the usefulness of this rat strain in the study of PE renal pathology. Nevertheless, we have shown novel transcriptomic/methylomics changes in rat models of healthy and complicated pregnancies. Key findings include pregnancy-induced activation of innate immune gene pathways in both rat strains, and rat strain x pregnancy interaction in the metabolism of cholesterol, fatty acids, and hormones such as vitamin D. Dysregulation of maternal renal response to pregnancy is responsible for the pronounced decreases in 1, 25-(OH)_2_-D levels in the maternal circulation. We conclude that the BN rat is a useful model to understand the mechanisms of placental insufficiency and the integrated response of the maternal organism.

## Supporting information

S1 Fig Rat cohorts with corresponding assays. Each cohort consisted of 4 groups of rats (n = 5 rats per group).
(TIF)

S2 Fig Pregnancy outcomes in rat studies A and B. Study A cohorts: A) Fetal and placental weights (the weights of all pups within a litter were averaged and counted as 1, n = 5 litters), B) Litter size. Study B cohorts: C) Maternal Heart Rates and D) Maternal Mean Arterial Blood Pressure were measured in anesthetized rats. Bars represent the mean +/- error (n = 5 rats/group). * p<0.05 NP versus P18; # p<0.05 BN compared with CD rats strain.
(TIF)

S3 Fig Sequencing quality of representative RNA-seq (A, C, E, G) and RRBS (B, D, F, G) reads. A and B, Phred quality scores at each position. The central red line is the median value. C and D, Per base sequence content after adaptor sequence removal. E and F, Sequence duplication levels. G and H, GC distribution over all sequences.
(TIF)

S4 Fig Metrics of sequence mapping. RNA-seq sequences were mapped to rat genome Rnor 6.0 using STAR. RRBS sequences were mapped to Rnor 6.0 methylation genome (prepared by Bismark) using Bowtie 2.
(TIF)

S5 Fig Pregnancy-induced upregulation of maternal renal IRF7 protein levels in both CD and BN rat strains. SDS-PAGE of total protein extracts (50μg) was followed by immunoblotting for IRF7 as explained under methods. A) Bar graph show the averages ± error (n = 5 rats/group). B) Representative immunoblots. *p<0.05 NP vs. P.
(TIF)

S1 Table SYBR green primers for qPCR. (DOCX)

S2 Table Primary antibodies used for western blotting. (DOCX)

S3 Table Transcriptome and methylome reads map summary. (XLSX)

S4 Table DEG and DMC lists. (XLSX)

S5 Table Pregnancy-induced canonical pathways and upstream regulators. (XLSX)

S6 Table Rat strain-dependent canonical pathways and upstream regulators. (XLSX)

S7 Table Integration of DEGs with DMRs. (XLSX)

## List of abbreviations

BN rats: Brown Norway rats
CD(SD) rats: Cesarean-derived Sprague-Dawley
CAR: constitutive androstane receptor
D: vitamin D
DEG: differentially expressed gene
DMR: differentially methylated region
FGR: fetal growth restriction
FLT1: fms-like tyrosine kinase-1
GFR: glomerular filtration rate
LXR: liver X receptor
NP: non-pregnant
P18: pregnant at day 18
PE: preeclampsia
PPAR: peroxisome proliferator-activated receptor
PRR: pattern recognition receptors
RXR: retinoid X receptor
VDR: vitamin D receptor
VEGF: vascular endothelial growth factor
