# Supplementary figures and images for: Renal functional, transcriptome, and methylome adaptations in pregnant Sprague Dawley and Brown Norway rats
Source: PLoS One. 2022 Jun 16;17(6):e0269792. doi: 10.1371/journal.pone.0269792 (PMC9202892; doi:10.1371/journal.pone.0269792)

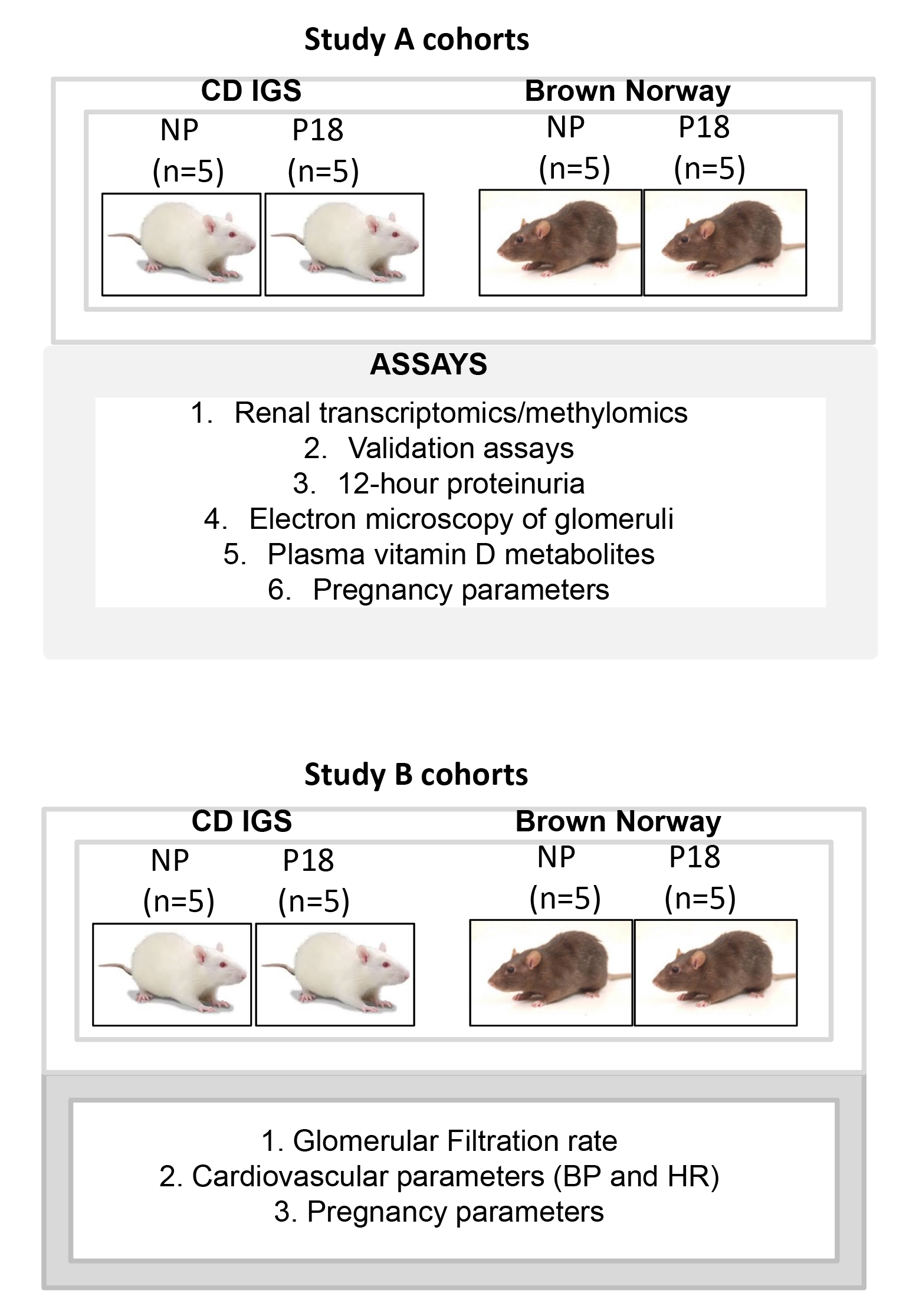

Supplement: S1 Fig — Each cohort consisted of 4 groups of rats (n = 5 rats per group). (TIF) [file pone.0269792.s001.tif]

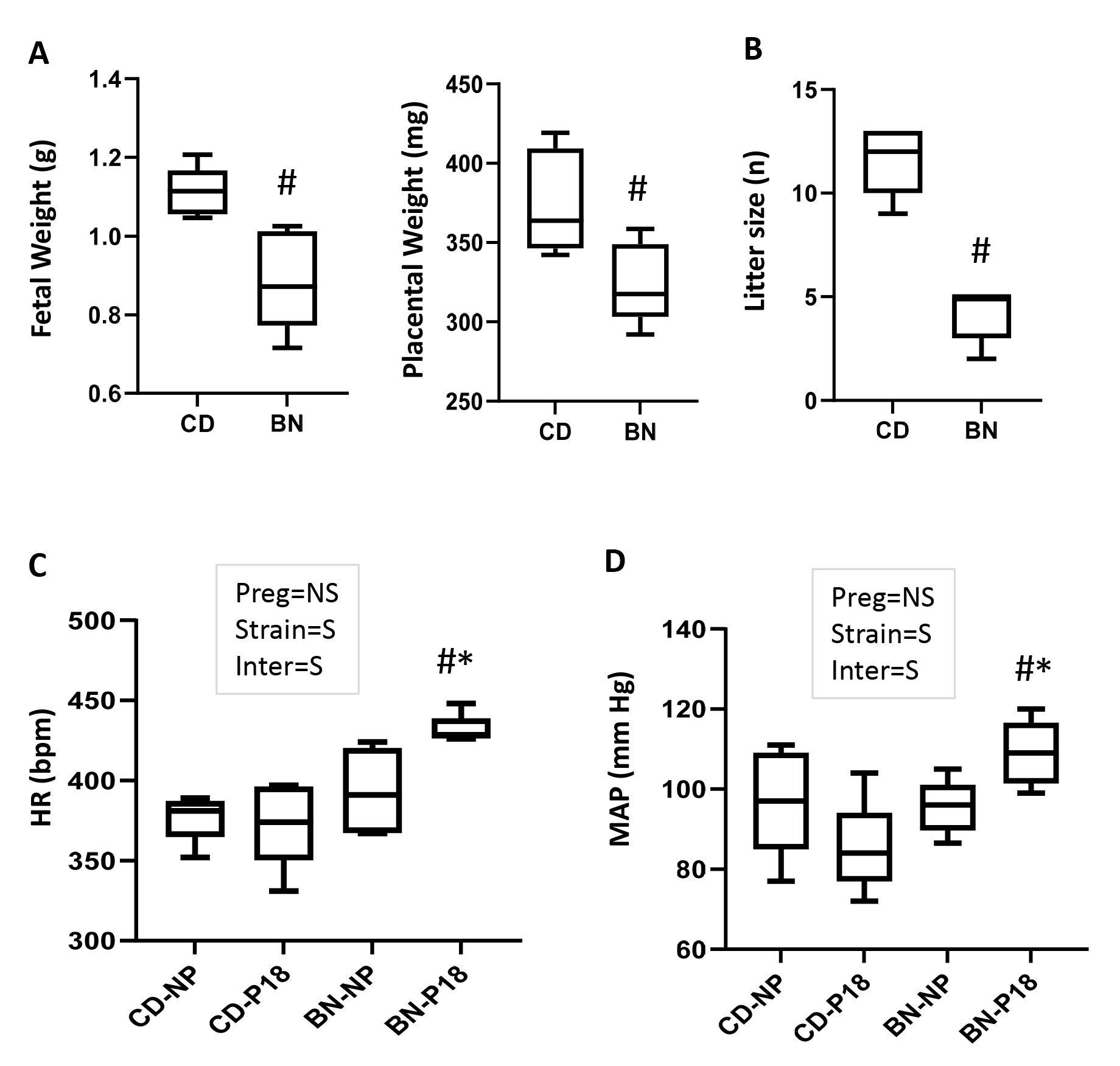

Supplement: S2 Fig — Pregnancy outcomes in rat studies A and B. Study A cohorts: A) Fetal and placental weights (the weights of all pups within a litter were averaged and counted as 1, n = 5 litters), B) Litter size. Study B cohorts: C) Maternal Heart Rates and D) Maternal Mean Arterial Blood Pressure were measured in anesthetized rats. Bars represent the mean +/- error (n = 5 rats/group). * p<0.05 NP versus P18; # p<0.05 BN compared with CD rats strain. (TIF) [file pone.0269792.s002.tif]

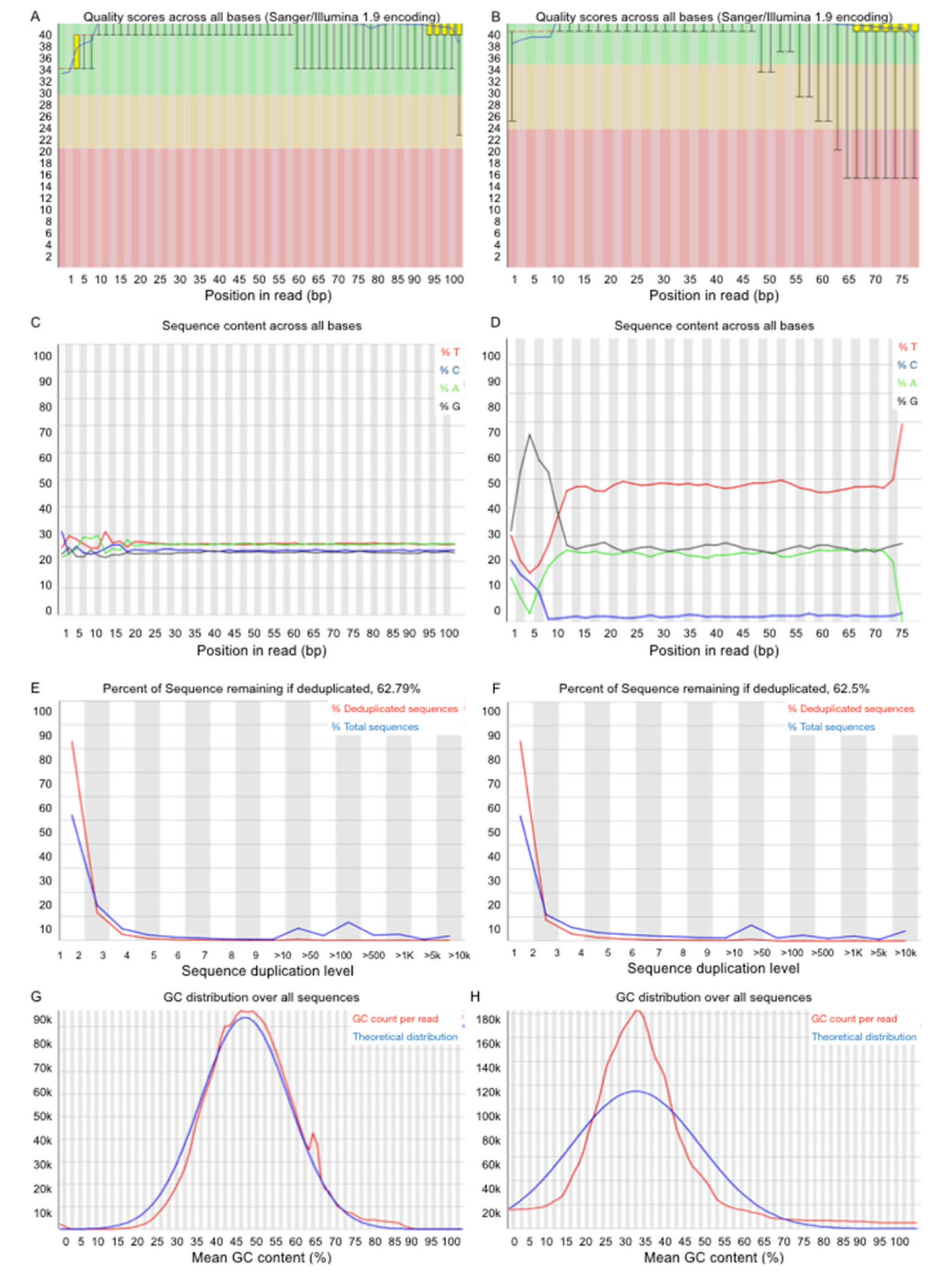

Supplement: S3 Fig — Sequencing quality of representative RNA-seq (A, C, E, G) and RRBS (B, D, F, G) reads. A and B, Phred quality scores at each position. The central red line is the median value. C and D, Per base sequence content after adaptor sequence removal. E and F, Sequence duplication levels. G and H, GC distribution over all sequences. (TIF) [file pone.0269792.s003.tif]

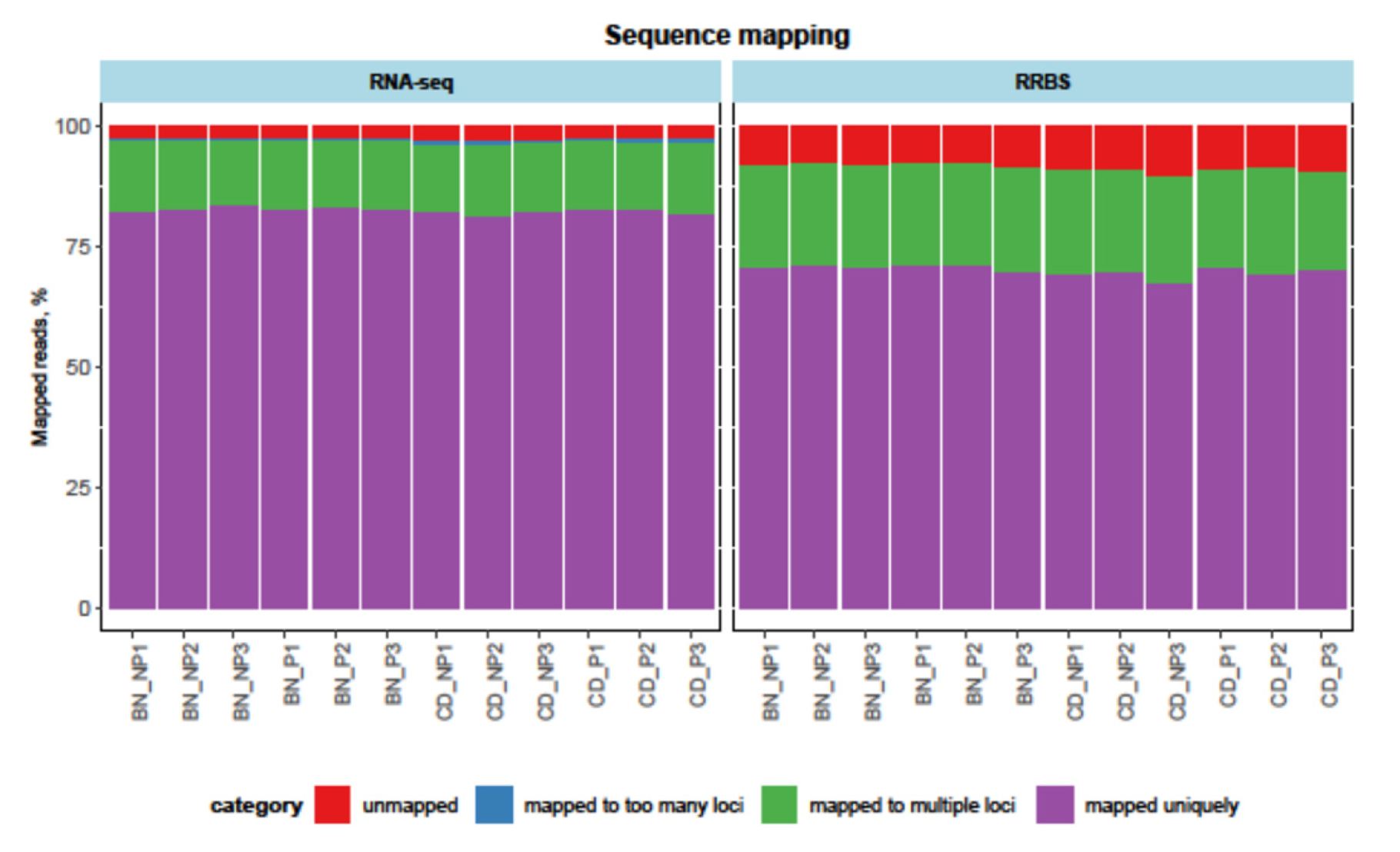

Supplement: S4 Fig — RNA-seq sequences were mapped to rat genome Rnor 6.0 using STAR. RRBS sequences were mapped to Rnor 6.0 methylation genome (prepared by Bismark) using Bowtie 2. (TIF) [file pone.0269792.s004.tif]

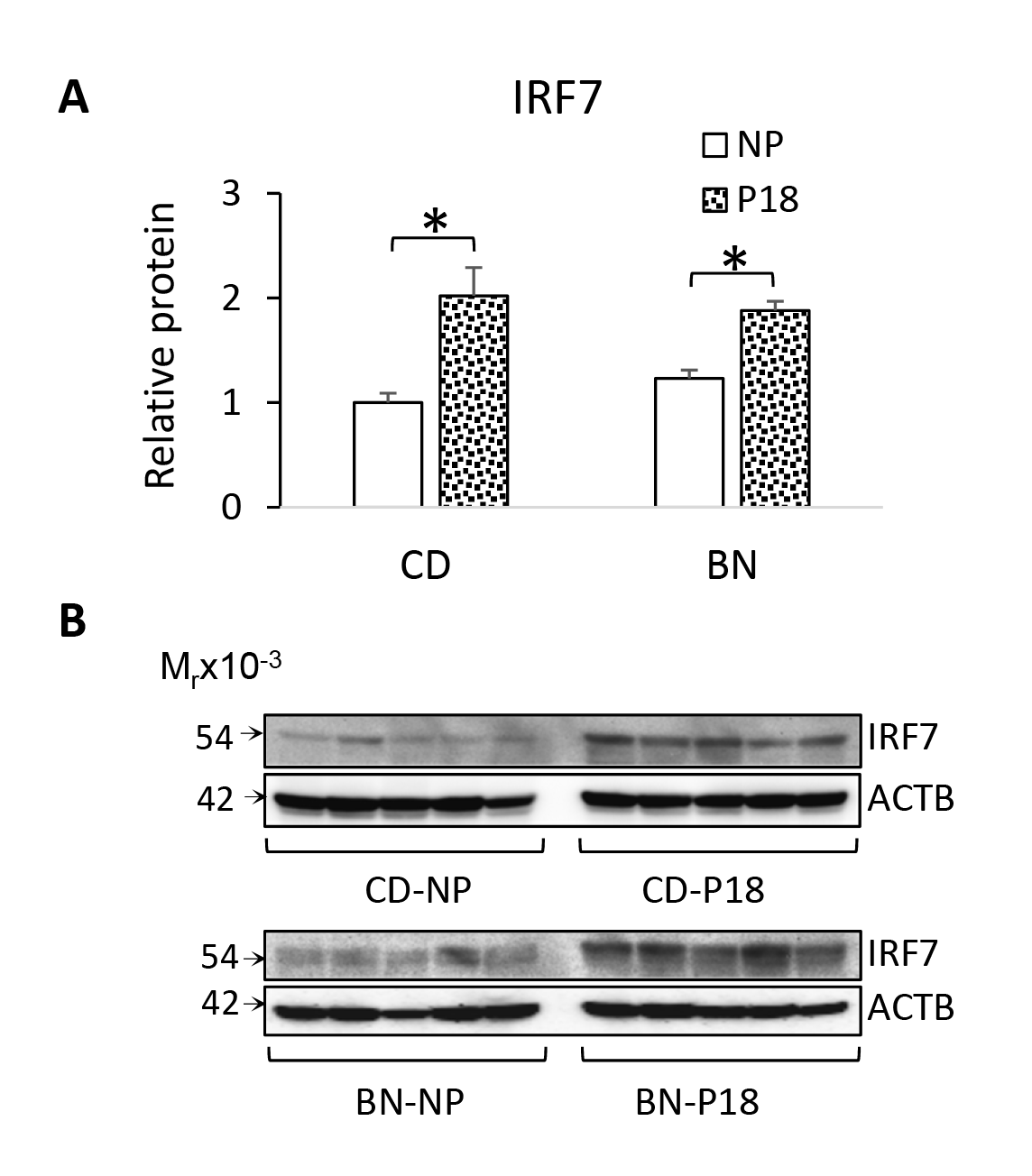

Supplement: S5 Fig — SDS-PAGE of total protein extracts (50μg) was followed by immunoblotting for IRF7 as explained under methods. A) Bar graph show the averages ± error (n = 5 rats/group). B) Representative immunoblots. *p<0.05 NP vs. P. (TIF) [file pone.0269792.s005.tif]
