# Supplementary material for: Renal functional, transcriptome, and methylome adaptations in pregnant Sprague Dawley and Brown Norway rats
Source: PLoS One. 2022 Jun 16;17(6):e0269792. doi: 10.1371/journal.pone.0269792 (PMC9202892; doi:10.1371/journal.pone.0269792)
Supplement: S1 Table — (DOCX) [file pone.0269792.s006.docx]

Supplementary Table 1: SYBR green primers for qPCR

| Gene | Primer sequences | Product size bp | Accession number |
| --- | --- | --- | --- |
| *Cyp27b1* | Fw 5’-CACCCATTTGCATCTCTTCC-3’  Rv 5’-GATGGATGCTCCTCTCAGGT-3’ | 187 | NM_053763.1 |
| *Cyp24a1* | Fw 5’-CAAACCTTGGAAAGCCTATCG-3’  Rv 5’-CTTTGCCACTCCTGTCCTT-3’ | 78 | NM_201635 |
| *Oas1a* | Fw 5’- GTGGTTCCCCAGTGAGTTCC -3’  Rv 5’- TCACTGTGTCAAGGTCCTGC -3’ | 140 | [NM_138913.1](https://www.ncbi.nlm.nih.gov/entrez/viewer.fcgi?db=nucleotide&id=25742703) |
| *Ifi27* | Fw 5’-ACTTACATCACTGGGTTTGGCT-3’  Rv 5’-GGAGGCAATAGAAGCCACCA-3’ | 93 | NM_203410.1 |
| *Fos* | Fw 5’-GAGCCGGTCAAGAACATTAGCA-3’  Rv 5’-GAAGGAACCAGACAGGTCCACAT-3’ | 136 | NM_022197.2 |
| *Mmp9* | Fw 5’- GATCCCCAGAGCGTTACTCG -3’  Rv 5’- GTTGTGGAAACTCACACGCC -3’ | 132 | [NM_031055.1](https://www.ncbi.nlm.nih.gov/entrez/viewer.fcgi?db=nucleotide&id=13591992) |
| *Nr1d1* | Fw 5’- CCGTGACCTTTCTCAGCACGA -3’  Rv 5’- GGAATTCTCCATTCCCGAGCG-3’ | 271 | [NM_001113422.1](https://www.ncbi.nlm.nih.gov/entrez/viewer.fcgi?db=nucleotide&id=164663888) |
| *Pigr* | Fw 5’-GGTCTATGGAGAAACTACAGCCA-3’  Rv 5’-CCAGGGTGGAGAATAGGACTTTG-3’ | 293 | NM_012723.4 |
| *Actb* | Fw 5’-GGCCAACCGTGAAAAGATGA-3’  Rv 5’-GACCAGAGGCATACAGGGACA-3’ | 101 | NM_031144.3 |
| *Mt1* | Fw 5’-CTCCTGCAAGAAGAGCTGCTG-3’  Rv 5’-GCAGCACTGTTCGTCACTTC-3’ | 124 | [NM_138826.4](https://www.ncbi.nlm.nih.gov/entrez/viewer.fcgi?db=nucleotide&id=82617664) |
| MeDIP *Mt1* | Fw 5’- GCAACGGTGTAAGCGACAAG -3’  Rv 5’- AACCGCCAACTGAGTGCAAA -3’ | 127 | NC_005118.4 |
| *Slc22a13* | Fw 5’-GGAACAGTACCACAAGGCGA-3’  Rv 5’-GTTTTGTAGGACCGTGGGGG-3’ | 156 | [NM_001126285.1](https://www.ncbi.nlm.nih.gov/nucleotide/187282170?report=gbwithparts) |
| MeDIP *Slc22a13* | Fw 5’-AAAGCCAACTCCTAGCTCCG-3’  Rv 5’-CAGGAGGTCAGCCAAGTCAC-3’ | 159 | NC_005107.4 |
