# Supplementary material for: Renal functional, transcriptome, and methylome adaptations in pregnant Sprague Dawley and Brown Norway rats
Source: PLoS One. 2022 Jun 16;17(6):e0269792. doi: 10.1371/journal.pone.0269792 (PMC9202892; doi:10.1371/journal.pone.0269792)
Supplement: S2 Table — (DOCX) [file pone.0269792.s007.docx]

Supplementary Table 2: Primary antibodies used for Western Blotting

| Antibody | Species | Dilution | Company | Catalog # |
| --- | --- | --- | --- | --- |
| IRF7 | Rabbit | 1:1000 | Cell Signaling Technology | D8V1J |
| PPARA | Mouse | 1:1000 | Novus Biologicals | NB300-537 |
| PDGF-B | Mouse | 1:200 | Santa Cruz Biotechnology | SC-365805 |
| NF-kB p65 (RELA) | Rabbit | 1:1000 | Cell Signaling Technology | D14E12 |
| β-Actin (ACTB) | Mouse | 1:5000 | Sigma Aldrich | A5441 |
| HSP90 | Rabbit | 1:2000 | Proteintech | 13171-1-AP |
| TBP | Mouse | 1:1000 | Abcam | ab51841 |
